# Neoadjuvant chemotherapy plus nivolumab with or without ipilimumab in operable non-small cell lung cancer: the phase 2 platform NEOSTAR trial

**DOI:** 10.1038/s41591-022-02189-0

**Published:** 2023-03-16

**Authors:** Tina Cascone, Cheuk H. Leung, Annikka Weissferdt, Apar Pataer, Brett W. Carter, Myrna C. B. Godoy, Hope Feldman, William N. William, Yuanxin Xi, Sreyashi Basu, Jing Jing Sun, Shalini S. Yadav, Frank R. Rojas Alvarez, Younghee Lee, Aditya K. Mishra, Lili Chen, Monika Pradhan, Haiping Guo, Ansam Sinjab, Nicolas Zhou, Marcelo V. Negrao, Xiuning Le, Carl M. Gay, Anne S. Tsao, Lauren Averett Byers, Mehmet Altan, Bonnie S. Glisson, Frank V. Fossella, Yasir Y. Elamin, George Blumenschein, Jianjun Zhang, Ferdinandos Skoulidis, Jia Wu, Reza J. Mehran, David C. Rice, Garrett L. Walsh, Wayne L. Hofstetter, Ravi Rajaram, Mara B. Antonoff, Junya Fujimoto, Luisa M. Solis, Edwin R. Parra, Cara Haymaker, Ignacio I. Wistuba, Stephen G. Swisher, Ara A. Vaporciyan, Heather Y. Lin, Jing Wang, Don L. Gibbons, J. Jack Lee, Nadim J. Ajami, Jennifer A. Wargo, James P. Allison, Padmanee Sharma, Humam Kadara, John V. Heymach, Boris Sepesi

**Affiliations:** 1 Department of Thoracic/Head and Neck Medical Oncology, The University of Texas MD Anderson Cancer Center, Houston, TX USA; 2 Department of Biostatistics, The University of Texas MD Anderson Cancer Center, Houston, TX USA; 3 Department of Pathology, The University of Texas MD Anderson Cancer Center, Houston, TX USA; 4 Department of Thoracic and Cardiovascular Surgery, The University of Texas MD Anderson Cancer Center, Houston, TX USA; 5 Department of Thoracic Imaging, The University of Texas MD Anderson Cancer Center, Houston, TX USA; 6 Department of Bioinformatics and Computational Biology, The University of Texas MD Anderson Cancer Center, Houston, TX USA; 7 The Immunotherapy Platform, The University of Texas MD Anderson Cancer Center, Houston, TX USA; 8 Department of Translational Molecular Pathology, The University of Texas MD Anderson Cancer Center, Houston, TX USA; 9 Platform for Innovative Microbiome and Translational Research (PRIME-TR), Department of Genomic Medicine, The University of Texas MD Anderson Cancer Center, Houston, TX USA; 10 Department of Imaging Physics, The University of Texas MD Anderson Cancer Center, Houston, TX USA; 11 Department of Surgical Oncology, The University of Texas MD Anderson Cancer Center, Houston, TX USA; 12 Department of Immunology, The University of Texas MD Anderson Cancer Center, Houston, TX USA; 13 Department of Genitourinary Medical Oncology, The University of Texas MD Anderson Cancer Center, Houston, TX USA; 14 Department of Cancer Biology, The University of Texas MD Anderson Cancer Center, Houston, TX USA; 15 Present Address: Hospital BP, a Beneficencia Portuguesa de Sao Paulo, Sao Paulo, Brazil

**Keywords:** Non-small-cell lung cancer, Translational research, Translational immunology, Tumour immunology, Phase II trials

## Abstract

Neoadjuvant ipilimumab + nivolumab (Ipi+Nivo) and nivolumab + chemotherapy (Nivo+CT) induce greater pathologic response rates than CT alone in patients with operable non-small cell lung cancer (NSCLC). The impact of adding ipilimumab to neoadjuvant Nivo+CT is unknown. Here we report the results and correlates of two arms of the phase 2 platform NEOSTAR trial testing neoadjuvant Nivo+CT and Ipi+Nivo+CT with major pathologic response (MPR) as the primary endpoint. MPR rates were 32.1% (7/22, 80% confidence interval (CI) 18.7–43.1%) in the Nivo+CT arm and 50% (11/22, 80% CI 34.6–61.1%) in the Ipi+Nivo+CT arm; the primary endpoint was met in both arms. In patients without known tumor *EGFR*/*ALK* alterations, MPR rates were 41.2% (7/17) and 62.5% (10/16) in the Nivo+CT and Ipi+Nivo+CT groups, respectively. No new safety signals were observed in either arm. Single-cell sequencing and multi-platform immune profiling (exploratory endpoints) underscored immune cell populations and phenotypes, including effector memory CD8^+^ T, B and myeloid cells and markers of tertiary lymphoid structures, that were preferentially increased in the Ipi+Nivo+CT cohort. Baseline fecal microbiota in patients with MPR were enriched with beneficial taxa, such as *Akkermansia*, and displayed reduced abundance of pro-inflammatory and pathogenic microbes. Neoadjuvant Ipi+Nivo+CT enhances pathologic responses and warrants further study in operable NSCLC. (ClinicalTrials.gov registration: NCT03158129.)

## Abstract (web summary)

The combination of neoadjuvant nivolumab, ipilimumab and chemotherapy showed promising efficacy in patients with resectable non-small cell lung cancer, with higher tumor immune cell infiltration and tertiary lymphoid structures after treatment compared with neoadjuvant nivolumab plus chemotherapy.

## Main

Immune checkpoint therapy has changed the treatment paradigm for patients with non-small cell lung cancer (NSCLC); however, until recently, much of the progress had been confined to the locally advanced and metastatic setting. Now, considerable effort is focused on how to best leverage immune checkpoint therapy for patients with resectable early-stage NSCLC and prevent post-operative tumor recurrence^1^, using adjuvant^2^ or neoadjuvant^3^ approaches targeting the PD-(L)1 axis. Neoadjuvant immunotherapy trials are based on the premise that an intact tumor immune microenvironment elicits the most robust responses to immune checkpoint inhibitors^1^. These trials have benefited by using major pathologic response (MPR) or complete pathologic response (pCR) as surrogate endpoints of long-term outcomes.

Studies of neoadjuvant single-agent anti-PD-(L)1 therapy have yielded MPR rates between 6.7% and 45%^4–8^. The addition of platinum-based chemotherapy to immunotherapy has proved promising^9^, with initial phase 2 studies producing MPR and pCR rates of 57–83% and 33–63%, respectively^10,11^. CheckMate-816 was the first large-scale phase 3 randomized trial to evaluate neoadjuvant nivolumab plus chemotherapy (Nivo+CT) versus chemotherapy (CT) alone in patients with resectable stage IB–IIIA NSCLC and demonstrated a pCR rate of 24.0% with Nivo+CT compared to 2.2% with CT alone, as well as improved event-free survival (EFS)^3^, which led to FDA approval of neoadjuvant Nivo+CT as the new standard of care for patients with resectable NSCLC.

Another strategy to enhance the efficacy of neoadjuvant anti-PD-(L)1 therapy is to combine it with the cytotoxic T-lymphocyte-associated protein (CTLA-4) immune checkpoint inhibitor ipilimumab (Ipi), given that the two inhibitors impact the immune system through two independent, and possibly complementary, mechanisms of action^12,13^. In the phase 2 randomized NEOSTAR study, we evaluated neoadjuvant Nivo or Nivo+Ipi followed by surgery in 44 patients with operable NSCLC^8^. We found that Nivo and Nivo+Ipi produced MPR rates of 22% and 38%, respectively. Addition of Ipi to Nivo also resulted in higher pCR rates, less viable tumor and enhanced tumor immune infiltration^8^.

**Extended Data Fig. 1:**
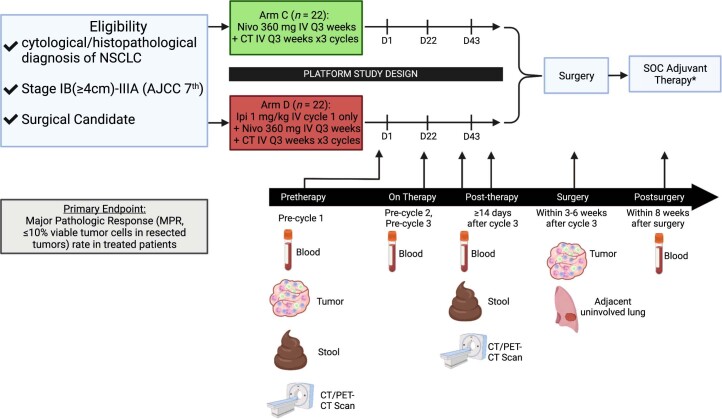
Trial schema. Patients with resectable, cytologically/histopathologically confirmed, clinical stage IB (≥4 cm)-IIIA (N2 single station) NSCLC were treated with neoadjuvant Nivo+CT for up to three cycles (arm C; D1, D22 and D43) or Ipi+Nivo+CT for up to three cycles (arm D; D1, D22 and D43; Ipi given on cycle 1 [D1] only), followed by surgical resection (within 3 to 6 weeks after the last cycle of therapy). *Standard of care adjuvant systemic therapy and/or postoperative radiation therapy were allowed at the discretion of the treating physicians. The primary endpoint of the trial was MPR, defined as ≤10% viable tumor in resected tumor specimens, in treated patients. Tumor samples were collected, when possible, pretherapy and at surgery, adjacent uninvolved (normal) lung tissues were also collected, where possible, at surgery. Stool samples were collected, where possible, pretherapy and post-therapy (prior to surgery). Longitudinal blood samples were collected, where possible, pretherapy, prior to cycle 2 and 3, post-therapy (prior to surgery) and within 8 weeks after surgery (post-surgery). NSCLC, non–small cell lung cancer; Nivo, nivolumab; Ipi, ipilimumab; CT, chemotherapy; D, day of therapy; CT, computer tomography scan; PET-CT, positron emission tomography-computer tomography scan, SOC, standard of care. BioRender (https://biorender.com) was used to generate portions of this Figure.

The randomized phase 2 NEOSTAR trial evolved into a platform trial of sequential, single-center, single-arm, phase 2 studies with a modular design using MPR in each individual arm as the primary endpoint, which was hypothesized to be greater than historical controls of neoadjuvant CT^14^. Here we report the primary efficacy results of NEOSTAR arm C evaluating neoadjuvant Nivo+CT and arm D testing neoadjuvant Ipi+Nivo+CT followed by surgical resection in patients with stage IB–IIIA NSCLC. Select secondary endpoints included radiological responses (RECIST version 1.1 (ref. ^15^)), pCR, toxicity, surgical resectability and perioperative morbidity/mortality, overall survival (OS) and EFS, in alignment with time-to-event analyses performed in other neoadjuvant studies^3,16^, and tissue immune infiltrate analysis. Exploratory endpoints included tumor molecular, immunological and fecal microbiome biomarkers (Extended Data Fig. 1).

## Results

### Patient baseline characteristics and treatment disposition

**Fig. 1:**
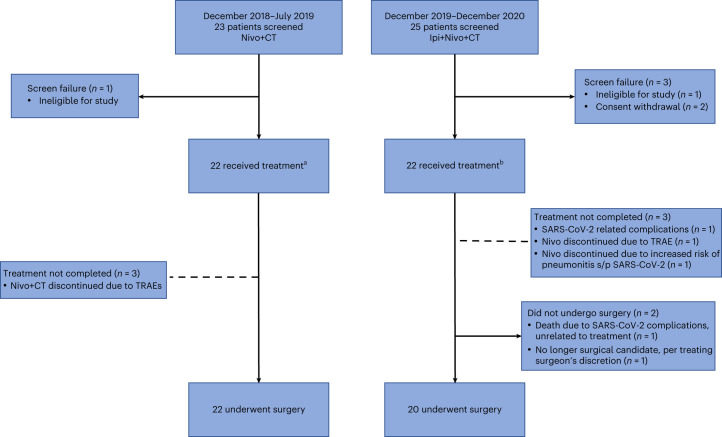
CONSORT flow diagram. Flow diagram depicts the disposition of patients throughout the phases of the study, including screening, neoadjuvant treatment and surgical resection. Reasons for screen failures, failure to complete planned neoadjuvant regimen and surgery not performed are shown. ^a^Eight patients required CT dose reduction. ^b^Seven patients required CT dose reduction, and four patients required platinum agent change.

Between 14 December 2018 and 22 July 2019, 23 patients were screened and 22 enrolled on the Nivo+CT treatment arm (Fig. 1). A full list of inclusion and exclusion criteria can be found in Methods. The baseline clinicopathological patient characteristics are shown in Table 1. All patients underwent baseline invasive mediastinal staging. Eleven (50%) patients had clinical stage IIIA (five with N2 disease, single station). Eighty-six percent (19/22) of patients completed the three planned cycles of neoadjuvant therapy, and 14% (3/22) received two cycles owing to treatment-related adverse events (TRAEs). Eight patients experienced CT dose reduction due to TRAEs. Between 30 December 2019 and 1 December 2020, 25 patients were screened and 22 enrolled on the Ipi+Nivo+CT treatment arm (Fig. 1). The baseline clinicopathological patient characteristics are shown in Table 1. All patients underwent invasive mediastinal staging. Thirteen (59%) patients presented with stage IIIA (nine with N2 disease, single station). Nineteen (86%) patients completed the planned three cycles of neoadjuvant therapies. Two patients discontinued nivolumab—one due to colitis possibly attributed to Ipi and Nivo (grade 3) after cycle one and one due to concern for increased risk of pneumonitis after cycle two and severe acute respiratory syndrome coronavirus 2 (SARS-CoV-2) infection. Neoadjuvant treatment was discontinued in one patient after cycle one due to death from SARS-CoV-2 infection-related complications (non-treatment related). Seven patients had CT dose reduction due to TRAEs. At the time of data analysis cutoff, 17 (77%) patients in the Nivo+CT arm and 15 (68%) patients in the Ipi+Nivo+CT arm had undergone ad hoc tumor molecular profiling (Supplementary Table 1). In the Nivo+CT arm, 53% had *TP53* mutations; 29% had *EGFR* mutations; 24% had *KRAS* mutations; and 6% had a *STK11* alteration. In the Ipi+Nivo+CT arm, 47% had *TP53* mutations; 33% had *EGFR* mutations; 33% had *KRAS* mutations; 7% had an *ALK* rearrangement; and 7% had a *STK11* alteration.

**Table 1:** Patient characteristics and treatment disposition

Variable		Nivo+CT (*n* = 22)	Ipi+Nivo+CT (*n* = 22)
Age—median (range), years		69.5 (45.6–79.3)	63.1 (39.4–77.5)
Age—number (%)	<65 years	10 (45)	13 (59)
	>65 years	12 (55)	9 (41)
Sex—number (%)	Female	12 (55)	7 (32)
	Male	10 (45)	15 (68)
Race—number (%)	Asian NOS	3 (14)	1 (5)
	Black	0 (0)	3 (14)
	White	19 (86)	18 (82)
Smoking status—number (%)	Never smoker	5 (23)	5 (23)
	Former smoker/current smoker	17 (77)	17 (77)
Stage—number (%)	Stage IB (≥4 cm) or II	11 (50)	9 (41)
	Stage IIIA	11 (50)	13 (59)
Histology—number (%)	Non-squamous	17 (77)	17 (77)
	Squamous	5 (23)	5 (23)
ECOG PS—number (%)	0	10 (45)	16 (73)
	1	12 (55)	6 (27)
Mediastinal staging—number (%)	EBUS	21 (95)	22 (100)
	Mediastinoscopy	1 (5)	0 (0)

EBUS, endobronchial ultrasound; NOS, not otherwise specified. Non-squamous includes adenocarcinoma, carcinoma with neuroendocrine features, NOS NSCLC, sarcomatoid carcinoma and large cell carcinoma.

### Pathologic tumor responses

**Fig. 2:**
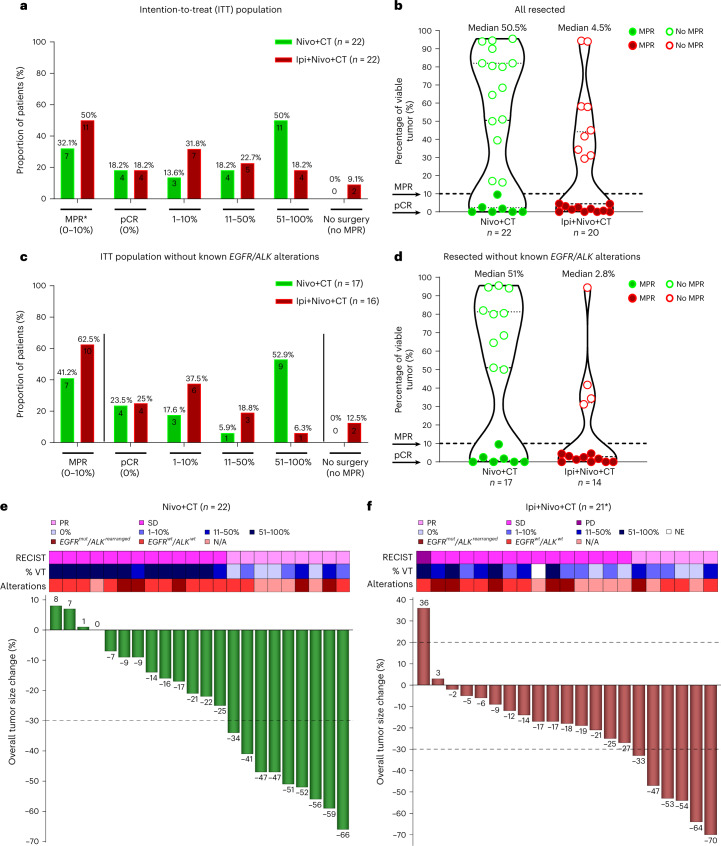
Pathologic and radiographic responses in patients treated with neoadjuvant Nivo+CT and Ipi+Nivo+CT. **a**, Proportion of patients with pathologic responses and percentage of viable tumor in the ITT population (Nivo+CT, *n* = 22; Ipi+Nivo+CT, *n* = 22). Primary endpoint: MPR (≤10% viable tumor cells) consists of pCR (0% viable tumor) and 1–10% viable tumor. *MPR rate was obtained from a UMVUE. **b**, Percentage of viable tumor in resected tumor specimens (Nivo+CT, *n* = 22; Ipi+Nivo+CT, *n* = 20). Median percentage of viable tumor: Nivo+CT 50.5% (range 0–95.5%) and Ipi+Nivo+CT 4.5% (range 0–94.4%). **c**, Proportion of patients with pathologic responses and percentage of viable tumor in ITT population without known tumor *EGFR/ALK* alterations (Nivo+CT, *n* = 17; Ipi+Nivo+CT, *n* = 16). **d**, Percentage of tumor in resected tumor specimens without known tumor *EGFR/ALK* alterations (Nivo+CT, *n* = 17; Ipi+Nivo+CT, *n* = 14). Median percentage of viable tumor: Nivo+CT 51% (range 0–95.5%) and Ipi+Nivo+CT 2.8% (range 0–94.4%). Data in **b** and **d** are presented as the median with minima, lower and upper quartiles and maxima using violin plots. The dashed line indicates the median; the dotted lines indicate the lower quartile and upper quartile values; and the top and bottom indicate the maxima and minima. The two arrows show percentage of viable tumor at MPR and pCR. The green filled and empty circles depict data from MPR and no MPR, respectively, in Nivo+CT patients, and the red filled and empty circles depict data from MPR and no MPR, respectively, in Ipi+Nivo+CT patients. **e**,**f**, The top panel shows the radiographic response by RECIST, percentage of viable tumor and select tumor molecular alterations, and the bottom panel shows the radiographic percentage change in overall tumor size from baseline in Nivo+CT (**e**) and Ipi+Nivo+CT (**f**). The dashed line at the 20% point depicts cutoff for PD. The dashed line at the −30% point depicts cutoff for PR. *One patient was not radiographically and pathologically evaluable due to death from SARS-CoV-2 infection-related complications (non-treatment related). VT, viable tumor; mut, mutant; wt, wild type; NE, not evaluable, N/A, not available.

**Extended Data Fig. 2:**
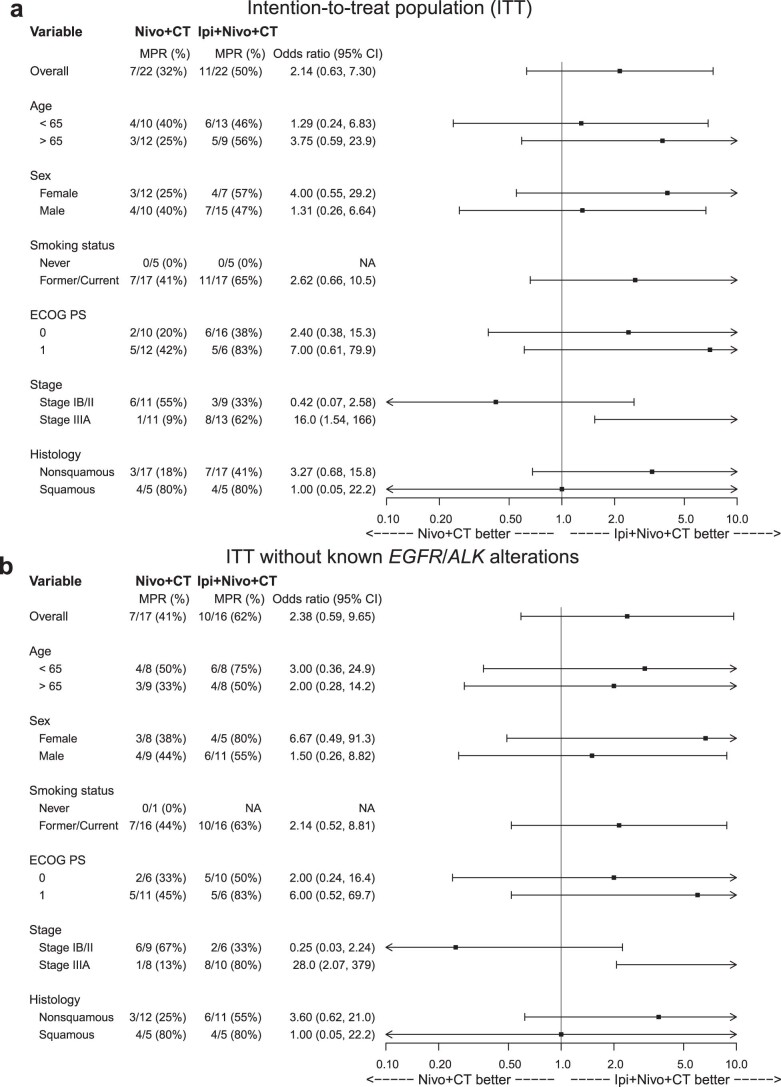
Impact of clinicopathological characteristics on efficacy of neoadjuvant Nivo+CT and Ipi+Nivo+CT. **a**, Forest plot of the odds ratio (95% confidence interval) to explore the association between treatment arm and MPR for each subgroup (Nivo+CT, *n* = 22; Ipi+Nivo+CT, *n* = 22). **b**, Forest plot of the odds ratio (95% confidence interval) to explore the association between treatment arm (Nivo+CT, *n* = 17; Ipi+Nivo+CT, *n* = 16) and MPR for each subgroup in patients without known tumor *EGFR/ALK* alterations. In both panels, the vertical reference lines at 1 indicate no difference between two treatment arms. The point estimates of odds ratios are represented by solid squares. The whiskers are the two lines that extend to the lower and upper bounds of the 95% confidence intervals. The lower and upper limits of the 95% confidence intervals are clipped at 0.1 and 10.0 to arrows. The odds ratios and 95% confidence interval are from univariate logistic regression. MPR, major pathologic response; Nivo, nivolumab; Ipi, ipilimumab; CT, chemotherapy; Never, never smoker; Former/Current, former/current smoker; ECOG, Eastern Cooperative Oncology Group; PS, performance status; Squamous, squamous cell carcinoma; Nonsquamous includes adenocarcinoma, carcinoma with neuroendocrine features, NOS NSCLC, sarcomatoid carcinoma, and large cell carcinoma; NA, not available.

In the intention-to-treat (ITT) population of 22 patients in the Nivo+CT arm, MPR occurred in seven patients (32.1%, 7/22, 80% confidence interval (CI) 18.7–43.1%, *P* = 0.036 for the statistical test against the assumed historical control of 15%), and this arm met the prespecified boundary of six responses to be considered efficacious; pCR occurred in four patients (18.2%, 4/22, 95% CI 5.2–40.3%) (Fig. 2a and Supplementary Table 2). All 22 treated patients underwent surgery on trial, and the median percentage of viable tumor was 50.5% (range 0–95.5%; Fig. 2b). In the ITT population of 22 patients in the Ipi+Nivo+CT arm, MPR occurred in 11 patients (50%, 11/22, 80% CI 34.6–61.1%, *P* = 0.00012 for the statistical test against the assumed historical control of 15%), also meeting the prespecified boundary of six responses to be considered efficacious; pCR occurred in four patients (18.2%, 4/22, 95% CI 5.2–40.3%) (Fig. 2a and Supplementary Table 2). Twenty patients (91%) underwent surgery on trial, and the median percentage of viable tumor was 4.5% (range 0–94.4%; Fig. 2b). In 20 resected patients, the MPR and pCR rates were 55% and 20%, respectively (Supplementary Table 3). The association between the treatment arm and MPR in subgroups of interest was explored (Extended Data Fig. 2a). Among the patients with stage IIIA disease, the odds of having MPR were 16.0 (95% CI 1.54–166) times higher in the Ipi+Nivo+CT arm than in the Nivo+CT arm. Similar results were obtained when analyses were performed in the population without known tumor *EGFR/ALK* alterations (Extended Data Fig. 2b). In both arms combined, the odds of having MPR among the former/current smokers was 23.6 (95% CI 1.11–498) times higher than among never smokers, and the odds of having MPR among patients with squamous histology was 9.60 (95% CI 1.73–53.4) times higher than among patients with non-squamous histologies (Supplementary Table 4).

**Extended Data Fig. 3:**
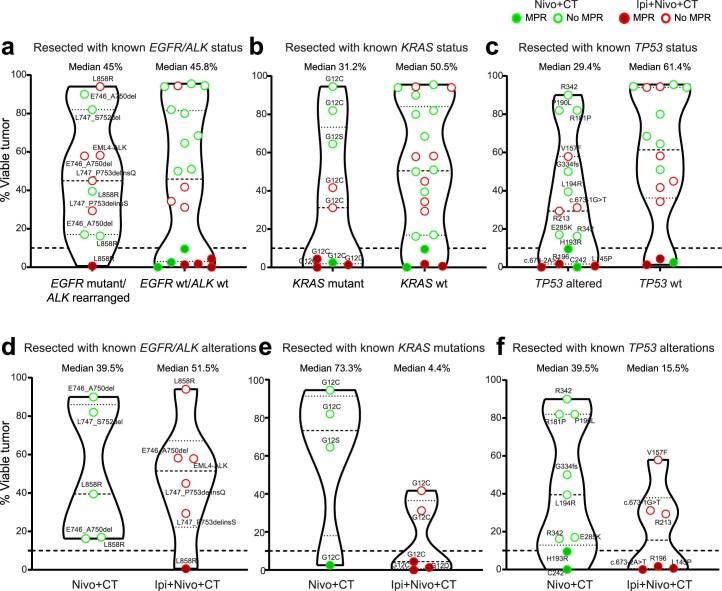
Pathological regression in resected patients with known tumor *EGFR/ALK*, *KRAS*, and *TP53* alterations. **a**-**c**, Comparison of the percentage of viable tumor in resected tumor specimens with: known *EGFR* mutant*/ALK* rearranged (*n* = 11) and *EGFR* wt*/ALK* wt (*n* = 20), median percentage of viable tumor: *EGFR* mutant*/ALK* rearranged 45% (range 0.6 – 94%), *EGFR* wt/*ALK* wt 45.8 (range 0 – 95.5%) (**a**); known *KRAS* mutant (*n* = 9) and *KRAS* wt (*n* = 22), median percentage of viable tumor: *KRAS* mutant 31.2% (range 0 – 94.5%), *KRAS* wt 50.5% (range 0 – 95.5%) (**b**); known *TP53* altered (*n* = 15) and *TP53* wt (*n* = 16), median percentage of viable tumor: *TP53* altered 29.4% (range 0 – 90%), *TP53* wt 61.4% (range 1.3 – 95.5%) (**c**). **d**-**f**, Percentage of viable tumor in resected tumor specimens with: known *EGFR* mutant*/ALK* rearranged tumors (Nivo+CT, *n* = 5; Ipi+Nivo+CT, *n* = 6), median percentage of viable tumor in Nivo+CT 39.5% (range 16.3 – 90%), in Ipi+Nivo+CT 51.5% (range 0.6 – 94%) (**d**); known *KRAS* mutant (Nivo+CT, *n* = 4; Ipi+Nivo+CT, *n* = 5), median percentage of viable tumor in Nivo+CT 73.3% (range 2.5 – 94.5%), in Ipi+Nivo+CT 4.4% (range 0 – 41.7%) (**e**); known *TP53* altered (Nivo+CT, *n* = 9; Ipi+Nivo+CT, *n* = 6), median percentage of viable tumor in Nivo+CT 39.5% (range 0 – 90%), in Ipi+Nivo+CT 15.5% (range 0 – 57.9%) (**f**). Tumor alterations are shown as amino acid change; the splice site variants c.673-1 G>T and c.673-2A>T are shown as codon change. One patient (who had tumor *TP53* alteration) was not included due to death from SARS-CoV-2 infection-related complications (non-treatment related) during neoadjuvant treatment. Dashed line at 10% point depicts cutoff for MPR. The green filled and empty circles depict data from MPR and no MPR, respectively, in Nivo+CT patients; the red filled and empty circles depict data from MPR and no MPR, respectively, in Ipi+Nivo+CT patients. Data are presented as median with minima, lower and upper quartiles, and maxima using violin plots. The dashed line indicates the median; the dotted lines indicate the lower quartile and upper quartile values; top and bottom indicate the maxima and minima. MPR, major pathologic response; Nivo, nivolumab; Ipi, ipilimumab; CT, chemotherapy; wt, wild type.

The MPR and pCR rates increased to 41.2% (7/17; 95% CI 18.4–67.1%) and 23.5% (4/17; 95% CI 6.8–49.9%), respectively, when patients with known tumor *EGFR* mutations and *ALK* rearrangements (*EGFR/ALK* alterations) were excluded in the Nivo+CT arm (Fig. 2c and Supplementary Table 5). The MPR and pCR rates increased to 62.5% (10/16; 95% CI 35.4–84.8%) and 25% (4/16; 95% CI 7.3–52.4%), respectively, when patients known to have these alterations were excluded in the Ipi+Nivo+CT arm (Fig. 2c and Supplementary Table 5). The median percentage of viable tumor in resected patients without known tumor *EGFR/ALK* alterations was 51% (range 0–95.5%) in the Nivo+CT arm (*n* = 17) compared with 2.8% (range 0–94.4%) in the Ipi+Nivo+CT arm (*n* = 14) (Fig. 2d). There were no notable differences in the median percentage of residual viable tumor in resected tumors harboring *EGFR/ALK* alterations compared with wild type (Extended Data Fig. 3a). However, we noted deeper median pathological regression in resected tumors harboring *KRAS* and *TP53* alterations compared with wild type (Extended Data Fig. 3b,c). Overall, there were no marked differences in the median percentage of viable tumor in resected tumors with *EGFR/ALK* alterations between the treatment arms (Extended Data Fig. 3d), whereas deeper median pathological regression was noted in resected tumors harboring *KRAS* and *TP53* alterations between the treatment arms (Extended Data Fig. 3e,f).

### Radiographic responses

In the Nivo+CT arm, radiographic partial responses (PRs) occurred in 41% (9/22) of patients, and 59% (13/22) of patients achieved stable disease (SD). None of the patients experienced progressive disease (PD) (Fig. 2e). There was a significantly greater reduction in overall tumor size from baseline to post-therapy in patients with MPR as compared to patients without MPR (*P* = 0.002; Supplementary Fig. 1a). In the Ipi+Nivo+CT arm, there were 21/22 radiographically evaluable patients due to one SARS-CoV-2 infection-related death while on neoadjuvant therapy. PR occurred in 29% (6/21) of evaluable patients (27% of ITT); SD was observed in 67% (14/21) of evaluable patients (64% of ITT); and one (5%) evaluable patient had radiographic PD (4.5% of ITT) (Fig. 2f). We also noted a significantly greater reduction in overall tumor size from baseline to post-therapy in patients with MPR as compared to patients without MPR in this treatment arm (Supplementary Fig. 1b; *P* = 0.041).

### Surgical therapy and perioperative outcomes

In the Nivo+CT arm, all 22 (100%) patients underwent planned surgical resection, and the R0 resection rate was 90% (20/22). Median time from the last dose of neoadjuvant therapy to operation was 33 days (range 23–138), with four (18%) operations being delayed due to TRAEs in three patients and pulmonary embolism in one patient. Lobectomy was performed in 17 (77.3%) patients, wedge in one (4.5%) patient, segmentectomy in two (9.1%) patients and pneumonectomy in two (9.1%) patients (Supplementary Fig. 2). The 30-day complication rate was 31.8% (7/22). The 30-day and 90-day mortality rates were 0%.

In the Ipi+Nivo+CT arm, 20/22 (91%) patients underwent planned operation with 18 (90%) lobectomies, of which one was sleeve and one bilobectomy. One (5%) patient underwent segmentectomy, and one (5%) patient underwent left pneumonectomy (Supplementary Fig. 2). The R0 resection rate was 95% (19/20). One patient died of SARS-CoV-2 infection-related complications (non-treatment related) after the first cycle of neoadjuvant therapy. Another patient was not resected, despite radiographic SD after completing neoadjuvant therapy, based on surgeon’s judgment because the tumor was abutting the left internal mammary artery to left anterior descending artery graft, and the patient’s exercise test performance declined. The median time to operation was 28.5 days (range 23–72), with two operations being delayed due to scheduling and a positive preoperative SARS-CoV-2 test requiring quarantine before surgery. The 30-day complication rate was 65% (13/20). The 30-day and 90-day mortality rates were 0%.

### Toxicity

All patients were included in the toxicity analysis (secondary endpoint) (Supplementary Table 6). All 22 patients in the Nivo+CT arm and 20 of 22 patients in the Ipi+Nivo+CT arm experienced TRAEs. In the Nivo+CT arm, 12 (55%) patients experienced grade (G) 1–2 TRAEs (nine G2 and three G1), and ten (45%) patients experienced G3–4 TRAEs (four G4 and six G3) by maximum grade. G4 TRAEs included hypercalcemia, hyponatremia and sepsis. In the Ipi+Nivo+CT arm, 16 patients (80%) experienced G1–2 TRAEs (eight G2 and eight G1), and four (20%) patients experienced G3–4 TRAEs (four G3) by maximum grade. G3 TRAEs included anemia, maculopapular rash, colitis and febrile neutropenia. Serious adverse events (SAEs) are reported in Supplementary Table 7.

### Survival outcomes

**Fig. 3:**
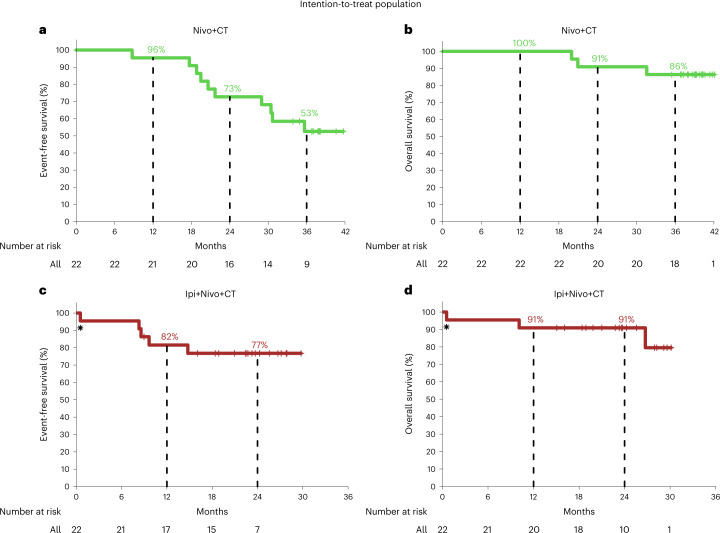
Survival outcomes in patients treated with neoadjuvant Nivo+CT and Ipi+Nivo+CT. **a**, Kaplan–Meier curve of EFS for the patients treated with neoadjuvant Nivo+CT (*n* = 22). Median EFS was not reached. Ten patients experienced recurrences 8.7 (died at 31.6 months), 17.7 (died at 20.0 months), 18.8 (died at 20.9 months), 19.5, 20.6, 21.7, 29.0, 30.4, 30.7 and 35.7 months after treatment initiation. **b**, Kaplan–Meier curve of OS for the patients treated with neoadjuvant Nivo+CT (*n* = 22). Median OS was not reached. Three patients died from complications related to recurrent lung cancer 20.0 months, 20.9 months and 31.6 months after treatment initiation. **c**, Kaplan–Meier curve of EFS for patients treated with neoadjuvant Ipi+Nivo+CT (*n* = 22). Median EFS was not reached. Four patients experienced recurrences 8.3, 8.6 (died at 26.7 months), 9.6 (died at 10.1 months) and 14.8 months after treatment initiation. *One patient died of SARS-CoV-2 infection-related complications (non-treatment related). **d**, Kaplan–Meier curve of OS for the patients treated with neoadjuvant Ipi+Nivo+CT (*n* = 22). Median OS was not reached. Two patients died from acute limb ischemia complications and lung cancer complications 10.1 months and 26.7 months after treatment initiation. *One patient died from SARS-CoV-2 infection-related complications (non-treatment related).

**Extended Data Fig. 4:**
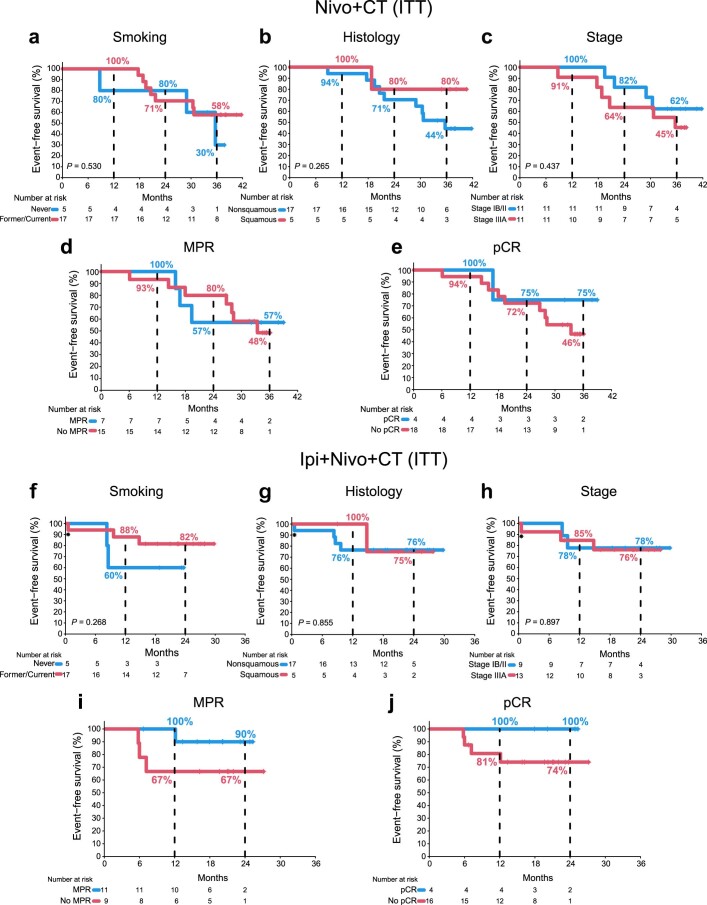
Impact of smoking status, histology, stage, MPR and pCR on EFS. **a**-**c**, Kaplan-Meier curves of EFS by smoking status (*n* = 22) (**a**), histology (*n* = 22) (**b**), and stage (*n* = 22) (**c**) in Nivo+CT arm. **d**-**e**, Kaplan-Meier curves of EFS by MPR (*n* = 22) (**d**) and pCR (*n* = 22) (**e**) using landmark analysis from the surgery date in Nivo+CT arm. **f**-**h**, Kaplan-Meier curves of EFS by smoking status (*n* = 22) (**f**), histology (*n* = 22) (**g**), and stage (*n* = 22) (**h**) in Ipi+Nivo+CT arm. *One patient died from SARS-CoV-2 infection-related complications (non-treatment related). **i**-**j**, Kaplan-Meier curves of EFS by MPR (*n* = 20) (**i**) and pCR (*n* = 20) (**j**) using landmark analysis from the surgery date in Ipi+Nivo+CT arm. Two-sided *P* values are from logrank test. ITT, intention-to-treat population; Nivo, nivolumab; Ipi, ipilimumab; CT, chemotherapy; EFS, event-free survival; Squamous, squamous cell carcinoma; Nonsquamous includes adenocarcinoma, carcinoma with neuroendocrine features, NOS NSCLC, sarcomatoid carcinoma, and large cell carcinoma; Never, never smoker; Former, former smoker; Former/Current, former/current smoker; MPR, major pathologic response; pCR, pathologic complete response.

**Extended Data Fig. 5:**
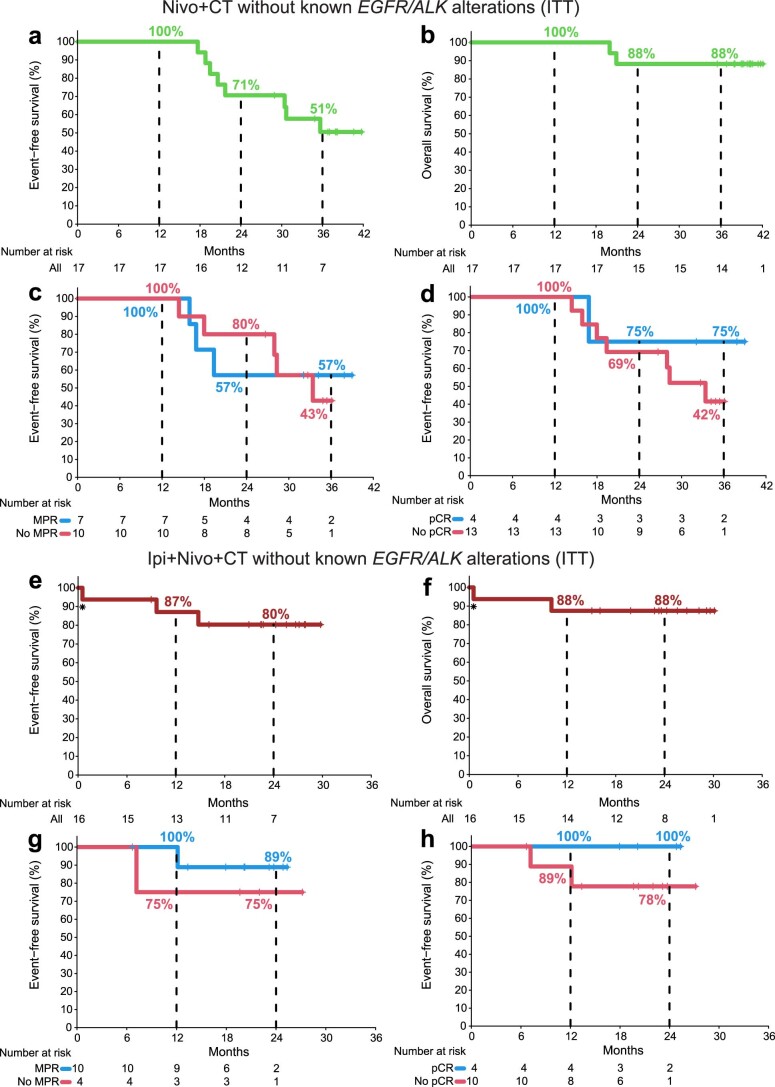
Survival outcomes in patients without known tumor *EGFR/ALK* alterations treated with neoadjuvant Nivo+CT and Ipi+Nivo+CT. **a**, Kaplan-Meier curve of EFS for the patients without known tumor *EGFR/ALK* alterations treated with Nivo+CT (*n* = 17). **b**, Kaplan-Meier curve of OS for the patients without known tumor *EGFR/ALK* alterations treated with Nivo+CT (*n* = 17). **c**, Kaplan-Meier curves of EFS by MPR status (*n* = 17) using landmark analysis from the surgery date in patients without known tumor *EGFR/ALK* alterations treated with Nivo+CT. **d**, Kaplan-Meier curve of EFS by pCR status (*n* = 17) using landmark analysis from the surgery date in patients without known tumor *EGFR/ALK* alterations treated with Nivo+CT. **e**, Kaplan-Meier curve of EFS for the patients without known tumor *EGFR/ALK* alterations treated with Ipi+Nivo+CT (*n* = 16). **f**, Kaplan-Meier curve of OS for the patients without known tumor *EGFR/ALK* alterations treated with Ipi+Nivo+CT (*n* = 16). In **e**-**f**, * indicates one patient who died from SARS-CoV-2 infection-related complications (non-treatment related). **g**, Kaplan-Meier curve of EFS by MPR status (*n* = 16) using landmark analysis from the surgery date in patients without known tumor *EGFR/ALK* alterations treated with Ipi+Nivo+CT. **h**, Kaplan-Meier curve of EFS by pCR status (*n* = 16) using landmark analysis from the surgery date in patients without known tumor *EGFR/ALK* alterations treated with Ipi+Nivo+CT. ITT, intention-to-treat population; Nivo, nivolumab; Ipi, ipilimumab; CT, chemotherapy; OS, overall survival; EFS, event-free survival; MPR, major pathologic response; pCR, pathologic complete response.

The last database check was on 18 July 2022. In the Nivo+CT arm, the median follow-up was 39.2 months. The median EFS and median OS were not reached (Fig. 3a,b). The EFS rate was 96% (95% CI 87–100%) at 12 months, 73% (95% CI 56–94%) at 24 months and 53% (95% CI 35–79%) at 36 months. Ten patients who had surgery experienced primary lung cancer-related recurrence from 8.7 months to 35.7 months after treatment initiation, and three of them later died. In the Ipi+Nivo+CT arm, the median follow-up was 24.0 months. The median EFS and median OS were not reached (Fig. 3c,d). The EFS rate was 82% (95% CI 67–100%) at 12 months and 77% (95% CI 61–97%) at 24 months. One patient died of treatment-unrelated complications from SARS-CoV-2. Four patients who had surgery experienced primary lung cancer-related recurrence from 8.3 months to 14.8 months, and two of them later died. In the Nivo+CT arm, analyses of EFS did not reveal notable differences with respect to smoking status, histology and clinical stage (Extended Data Fig. 4a–c). Landmark EFS analyses showed that any lung cancer-related recurrence occurred in 42% (3/7) of MPR versus 47% (7/15) of no MPR patients and in 25% (1/4) of pCR versus 50% (9/18) of no-pCR patients (Extended Data Fig. 4d,e). In the Ipi+Nivo+CT arm, analyses of EFS did not show notable differences with respect to smoking status, histology and clinical stage (Extended Data Fig. 4f–h). Landmark EFS analyses revealed that any lung cancer-related recurrence occurred in 9% (1/11) of MPR patients versus 33% (3/9) of no MPR patients and in 0% (0/4) of pCR patients versus 25% (4/16) of no-pCR patients (Extended Data Fig. 4i,j). The EFS, OS and landmark EFS analyses of patients without known tumor *EGFR*/*ALK* alterations are shown in Extended Data Fig. 5.

### Single-cell RNA sequencing

**Fig. 4:**
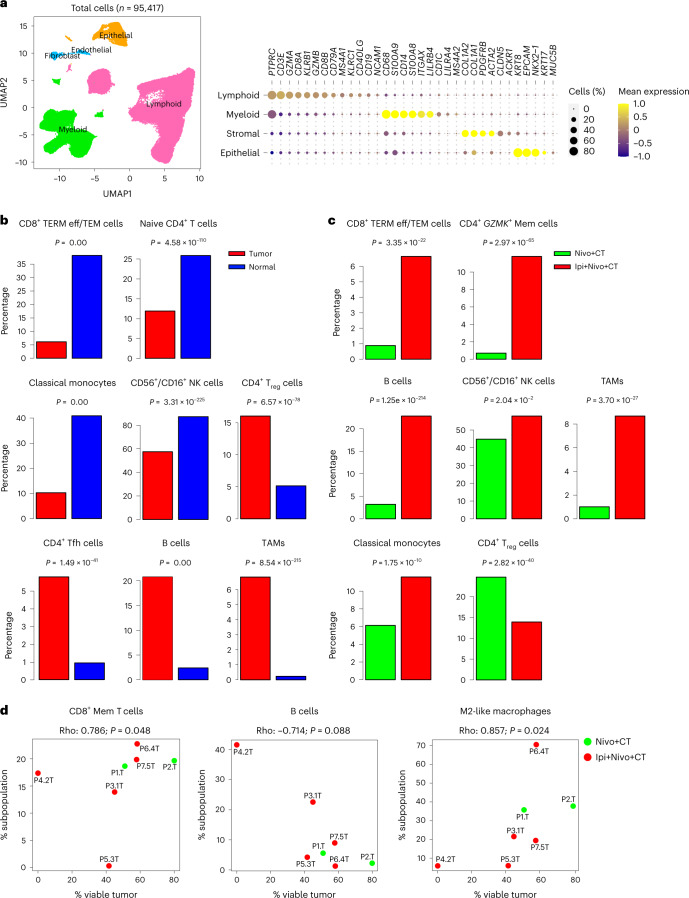
Single-cell expression analysis of resected tumors and uninvolved normal lung tissues from patients treated with neoadjuvant Nivo+CT and Ipi+Nivo+CT. scRNA-seq analysis was performed on matched NSCLCs and uninvolved normal lung tissues from patients treated with Nivo+CT (*n* = 2) and Ipi+Nivo+CT (*n* = 5). scRNA-seq was also performed on an LN sample from a patient treated with Nivo+CT. **a**, Left: UMAP visualization of 95,417 high-quality and non-cycling cells after clustering. Clusters are color-coded by major cell lineage: lymphoid, myeloid, epithelial and stromal (fibroblasts and endothelial cells). Right: bubble plot showing mean expression and abundance of marker genes that are differentially expressed among the four major lineage groups. **b**, Fractions of the indicated cell subsets from their respective lineages were computed in tumors (red bars) and normal tissues (blue bars) as such: CD8^+^ TERM eff/TEM from CD8^+^ T cells; naive CD4^+^ T cells, T_reg_ cells and Tfh cells from all CD4^+^ T cells; B cells from lymphoid cells; classical monocytes and TAMs from myeloid cells; and *NCAM1*^+^/*FCGR3A*^+^CD56^+^/CD16^+^ NK cells from all innate lymphoid cells. Fractions of the indicated cell subsets were then statistically compared between matched tumor and normal tissues from all seven patients. *P* values are from two-sided proportion test. **c**, Fractions of the indicated cell subsets from their respective lineages were computed in tumors from Nivo+CT (green bars) and tumors from Ipi+Nivo+CT (red bars), as in **b**, and were then statistically compared between tumors from both treatment groups. *P* values are from two-sided proportion test. **d**, Correlation plots between fractions of the indicated cell subpopulations and the percentage of remaining viable tumor at the time of surgical resection. Fractions were computed in the manner described above: CD8^+^ memory T cells (CD8^+^ Mem) from all CD8^+^ T cells, B cells from all lymphoid cells and M2-like macrophages from all myeloid cells. Correlation coefficients were computed using Spearman’s correlation. *P* values were computed by two-sided Spearman’s correlation test. Source data for **d** are provided in Supplementary Table 8.

**Extended Data Fig. 6:**
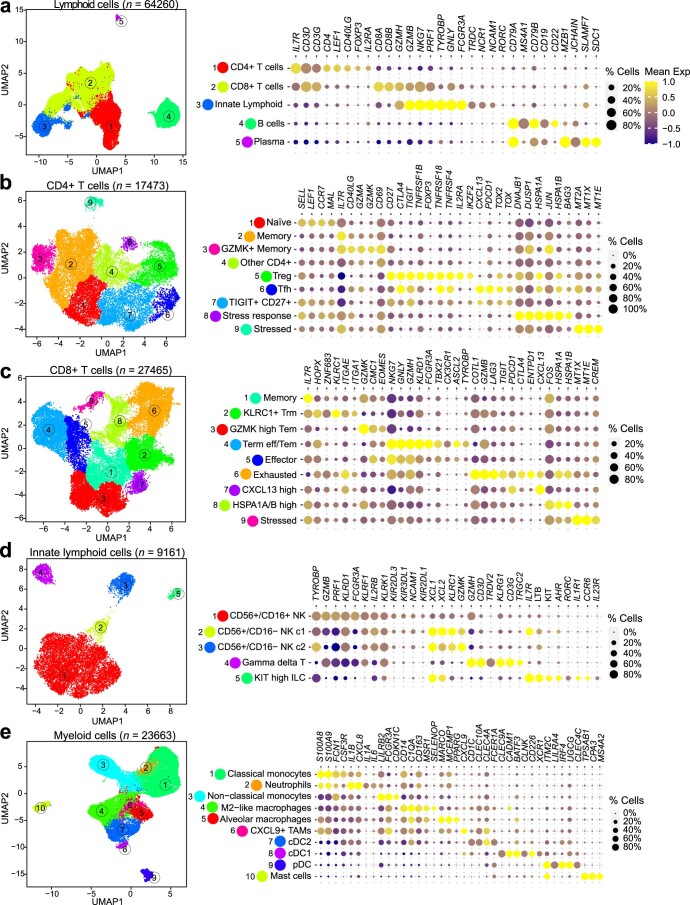
Single-cell analysis of lymphoid and myeloid cell populations in tissues from patients treated with neoadjuvant Nivo+CT and Ipi+Nivo+CT. **a**, Visualization of 64,260 lymphoid cells following clustering and color-coded by lymphoid lineages (CD4+T, CD8+ T, innate lymphoid, B, and plasma). Visualization of CD4+ T (**b**; *n* = 17,473), CD8+ T (**c**, *n* = 27,465), and innate lymphoid cells (**d**, *n* = 9,161) following clustering and color-coded by sublineages. **e**, Visualization of 23,663 myeloid cells following clustering and color-coded by lineages. All right panels: Bubble plots showing mean expression and abundance of marker genes that are differentially expressed among lymphoid cells (**a**), lymphoid subsets (**b-d**), and myeloid (**e**) cells based on lineage or sublineage.

We performed single-cell RNA sequencing (scRNA-seq) of cells derived from seven paired tumor and normal (tumor-uninvolved) tissues—two from the Nivo+CT arm and five from the Ipi+Nivo+CT arm (Supplementary Fig. 3). We also sequenced cells from an involved lymph node (LN) from one patient in the Nivo+CT arm. Pathologic response attributes and molecular characteristics of patients whose tissues underwent scRNA-seq are shown in Supplementary Table 8. After quality control and filtering of low-quality cells (Methods), we studied 97,943 high-quality cells from the 15 samples (Supplementary Fig. 4a–e). These comprised non-cycling subsets of major lineages (*n* = 95,417 cells; Fig. 4a), including stromal (endothelial and fibroblasts), epithelial, lymphoid and myeloid cells. Cycling cells (*n* = 2,526) originated from multiple lineages, mostly lymphoid and myeloid (Supplementary Fig. 4f). We identified different subsets within the lymphoid (*n* = 64,260 cells), including CD4^+^, CD8^+^ T cells and innate cells, and myeloid (*n* = 23,663 cells) compartments (Extended Data Fig. 6). Fractions of CD8^+^ terminally differentiated effector/effector memory (TERM eff/TEM) T cells, naive CD4^+^ T cells, monocytes and *NCAM1*^+^/*FCGR3A*^+^ natural killer (NK) cells were significantly decreased in tumors relative to uninvolved lung tissues (all *P* < 0.0001; Fig. 4b). Conversely, fractions of regulatory T (T_reg_) cells, T follicular helper (Tfh) cells, B cells and *CXCL9*^+^ tumor-associated macrophages (TAMs) were largely increased in tumors (all *P* < 0.0001; Fig. 4b).

Fractions of CD8^+^ TERM eff/TEM, CD4^+^ *GZMK*^+^ memory, B, NK (*NCAM1*^+^/*FCGR3A*^+^) cells, *CXCL9*^+^ TAMs as well as monocytes were markedly increased in tumors from the Ipi+Nivo+CT group relative to the Nivo+CT arm (all *P* < 0.0001; Fig. 4c). Conversely, fractions of T_reg_ cells were evidently decreased in tumors from patients treated with Ipi+Nivo+CT relative to those from patients treated with Nivo+CT (*P* < 0.0001; Fig. 4c). Additionally, CD8^+^ memory T cells (*R* = 0.786; *P* = 0.048) and M2-like macrophages (*R* = 0.857; *P* = 0.024) were positively correlated with the percentage of viable tumor (Fig. 4d), whereas an inverse trend was noted for B cells (*R* = −0.714; *P* = 0.088) (Fig. 4d). We compared gene expression changes within specific cell subsets between Nivo+CT and Ipi+Nivo+CT tumors and found increased features of immunosuppression in immune cell subsets from Nivo+CT tumors, including elevated levels of *CTLA4*, *LAG3* and *IL2RA* in T_reg_ cells (Supplementary Fig. 5a). We also noted significantly increased levels of *CD24* and *IGHA1* in B cells from Nivo+CT relative to Ipi+Nivo+CT tumors (Supplementary Fig. 5b). On that theme, Nivo+CT-treated tumors showed increased expression of *CXCL13* in both memory and exhausted CD8^+^ T cell subsets compared to that in Ipi+Nivo+CT-treated tumors (Supplementary Fig. 5c). We could not find marked changes in immune cell compositions and fractions based on major variables, such as smoking, genomic alterations and MPR, which may be due to the relatively small number of cases analyzed by scRNA-seq. Also, the seven tumors studied by scRNA-seq each exhibited distinct mutational changes (Supplementary Table 8). Nonetheless, our single-cell analyses underscored overall enhanced anti-tumor and reduced immunosuppressive phenotypes in patients treated with Ipi+Nivo+CT compared with those in the Nivo+CT arm.

### NanoString analysis of resected tumors

**Extended Data Fig. 7:**
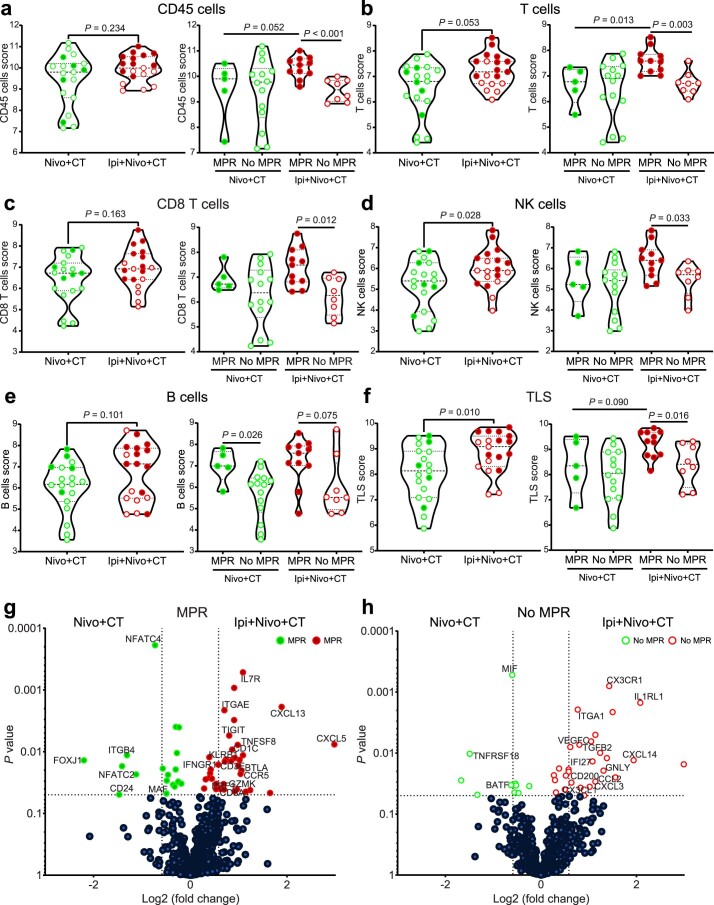
Composition of immune infiltrates by NanoString analysis in tumors resected after neoadjuvant Nivo+CT and Ipi+Nivo+CT. NanoString gene expression analysis was performed on post-treatment tumor tissues from patients treated with Nivo+CT (*n* = 19) and Ipi+Nivo+CT (*n* = 19). **a–f**, Violin plots showing the distribution of CD45+ (**a**), T cells (**b**), CD8+ T cells (**c**), NK cells (**d**), B cells (**e**), and TLS (**f**) scores (log2 normalized counts) in resected tumors by treatment arm (left panels) and by treatment arm and MPR (right panels). The TLS signature score is derived from the median expression of *CCL19, CCL21, CXCL13, CCR7, SELL, LAMP3, CXCR4, CD86*, and *BCL6* genes. The green filled and empty circles depict data from MPR and no MPR, respectively, in Nivo+CT patients, and the red filled and empty circles depict data from MPR and no MPR, respectively, in Ipi+Nivo+CT patients. Data are presented as the median with minima, lower and upper quartiles, and maxima using violin plots. The dashed line indicates the median; the dotted lines indicate the lower quartile and upper quartile values; the top and bottom indicate the maxima and minima. **g**, Differential expression of genes between Nivo+CT and Ipi+Nivo+CT in MPR samples are illustrated as a volcano plot. The green filled circles depict significantly upregulated genes in Nivo+CT compared to Ipi+Nivo+CT. The red filled circles depict significantly upregulated genes in Ipi+Nivo+CT compared to Nivo+CT. **h**, Differential expression of genes between Nivo+CT and Ipi+Nivo+CT in no MPR samples are illustrated as a volcano plot. The green empty circles depict significantly upregulated genes in Nivo+CT compared to Ipi+Nivo+CT. The red empty circles depict significantly upregulated genes in Ipi+Nivo+CT compared to Nivo+CT. Two-sided *P* values are from Wilcoxon rank-sum test in the left panels in **a** and **b** and the right panels in **a**-**f** and two-sided *P* values are from unpaired t-test in in the left panels in **c**-**f**. The exact two-sided *P* value in the right panel in **a** is 0.000793840. Two-sided *P* values are from Welch’s t-test in **g**-**h**. Nivo, nivolumab; Ipi, ipilimumab; CT, chemotherapy; MPR, major pathologic response. Source data is provided as Source Data file. Source data

To further characterize the immune composition of tumors treated with neoadjuvant chemoimmunotherapy and evaluate the impact of Ipi on the phenotype of tumor-infiltrating immune populations, we performed gene expression analysis by NanoString of resected tumors from patients treated with Nivo+CT and Ipi+Nivo+CT. We observed overall favorable immunological changes in tumors from the Ipi+Nivo+CT arm. Cell type scores for immune cells (CD45^+^), T cells, CD8^+^ T cells, NK cells, B cells, cytotoxic cells and macrophages were all greater in tumors resected from patients treated with Ipi+Nivo+CT compared to those treated with Nivo+CT (Extended Data Fig. 7a–e and Supplementary Fig. 6a,b, left panels). Signature scores of tertiary lymphoid structures (TLSs) were also significantly higher in tumors from patients treated with Ipi+Nivo+CT compared to Nivo+CT (Extended Data Fig. 7f and Supplementary Fig. 6c, left panels). The effect of combining Ipi with Nivo+CT was more evident when we segregated the samples based on treatment response. Pathologic responders (MPR) to Ipi+Nivo+CT had higher infiltration of CD45^+^ immune cells, including T cells, CD8^+^ T cells, NK cells, B cells, cytotoxic cells and macrophages compared to non-responders (no MPR) (Extended Data Fig. 7a–e and Supplementary Fig. 6a,b, right panels). Moreover, we observed a higher TLS gene signature score in MPR patients compared to no MPR patients (Extended Data Fig. 7f and Supplementary Fig. 6c, right panels).

Analysis of differentially expressed genes in responders (MPR) to Ipi+Nivo+CT compared to Nivo+CT showed an enrichment of genes associated with TLS formation, cytotoxic molecules and memory T cell markers, all of which have been shown to be associated with a favorable clinical outcome in patients with cancer treated with immune checkpoint therapies (Extended Data Fig. 7g). In contrast, non-responders (no MPR) to Ipi+Nivo+CT had significantly higher expression of immune genes associated with M2-like macrophages and other immunosuppressive genes compared to non-responders to Nivo+CT (Extended Data Fig. 7h). To investigate the potential impact of select tumor molecular alterations on the immune profiles of tumors treated with neoadjuvant therapy, we analyzed the immune scores in resected tumors by *EGFR* mutations/*ALK* rearrangements, *KRAS* mutations and *TP53* alterations as compared to wild-type tumors. We found no significant changes in NanoString-based immune scores between treated tumors harboring *EGFR/ALK*, *KRAS* or *TP5*3 alterations and their respective wild-type counterparts (Supplementary Tables 9, 10 and 11, respectively). Together, these results indicate that addition of Ipi to Nivo+CT leads to favorable immunological changes compared to Nivo+CT, and these changes are even more pronounced in patients who achieve MPR.

### Additional tissue immunological analyses

**Extended Data Fig. 8:**
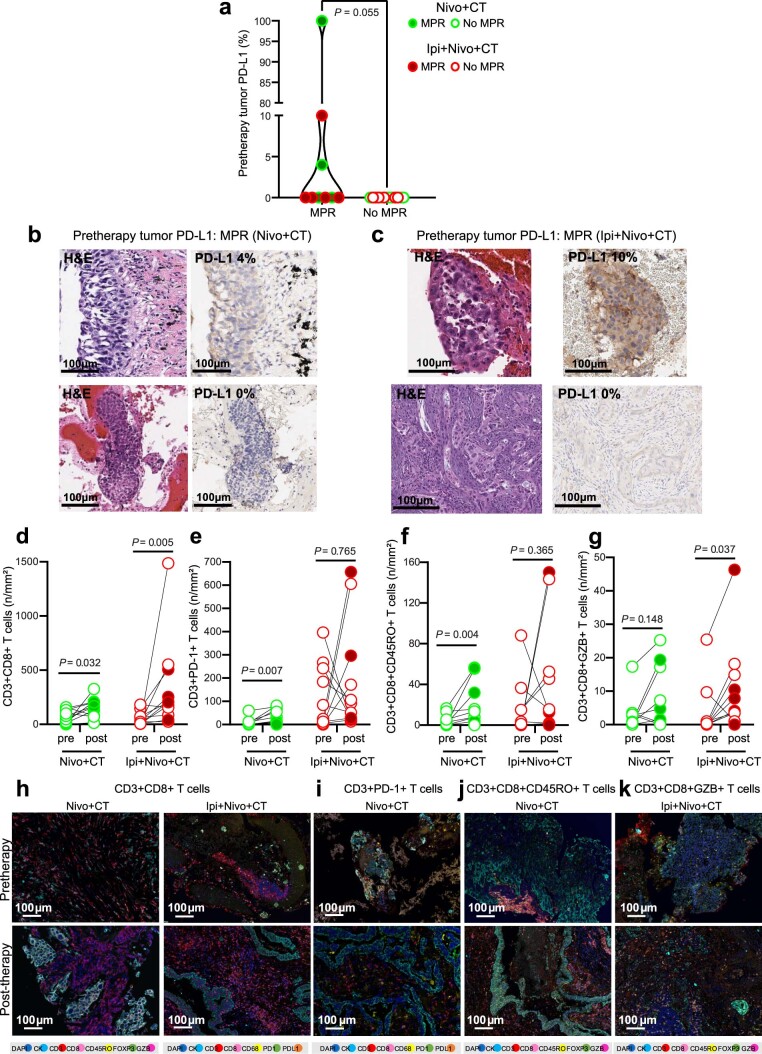
Tumor PD-L1 expression and immune population changes in tissues treated with neoadjuvant Nivo+CT and Ipi+Nivo+CT. **a**, Pretherapy tumor PD-L1 IHC membranous expression (%) in malignant cells from responders and nonresponders treated with Nivo+CT and Ipi+Nivo+CT by MPR status (MPR vs. no MPR, *n* = 9 vs. *n* = 13). Data is presented as the median with minima, lower and upper quartiles, and maxima using violin plots. Individual data points are shown; the dashed line shows the median value, and dotted lines show lower quartile and upper quartile values; the top and bottom of the violin plots indicate the maxima and minima. Two-sided *P* value is from Wilcoxon’s rank-sum test. **b**, **c**, Examples of hematoxylin and eosin (H&E) micrographs (left panels) of pretherapy tumors from patients with MPR in Nivo+CT (**b**) and in Ipi+Nivo+CT (**c**) arms with pretherapy PD-L1 expression (right and top panel) or without PD-L1 expression (right and bottom panel) in malignant cells. Experiments and scorings related to the presented micrographs were conducted once. **d**-**g**, Quantification of CD3+CD8+ T cells (panel 1) (**d**), CD3+PD-1+ T cells (panel 1) (**e**), CD3+CD8+CD45RO+ T cells (panel 2) (**f**), and CD3+CD8+GZB+ T cells (panel 2) (**g**) densities (no. per mm^2^) by mIF staining in paired pretherapy and post-therapy tumor samples in Nivo+CT (*n* = 11) and Ipi+Nivo+CT (*n* = 11) groups. Two-sided *P* value is from Wilcoxon’s signed-rank test. **h**-**k**, Examples of micrographs of mIF staining of pretherapy and post-therapy CD3+CD8+ T cells (panel 2 and 1) (**h**), CD3+PD-1+ T cells (panel 1) (**i**), CD3+CD8+CD45RO+ T cells (panel 2) (**j**), and CD3+CD8+GZB+ T cells (panel 2) (**k**) in tumor samples in Nivo+CT and Ipi+Nivo+CT arms. Experiments and scoring related the presented micrographs were conducted once. The green filled and empty circles depict data from MPR and no MPR, respectively, in Nivo+CT patients, and the red filled and empty circles depict data from MPR and no MPR, respectively, in Ipi+Nivo+CT patients. Nivo, nivolumab; Ipi, ipilimumab; CT, chemotherapy; MPR, major pathologic response. Source data for panels **a, d-g** is provided as Source Data file. Source data

The distribution of baseline PD-L1 expression by immunohistochemistry (IHC) in tumor cells according to MPR and treatment arm is depicted in Extended Data Fig. 8a. Responses were seen in patients with PD-L1-negative and PD-L1-positive tumors in both treatment arms (Extended Data Fig. 8a–c). A numerically higher proportion of patients with negative PD-L1 tumors experienced MPR in the Ipi+Nivo+CT group (40%, 4/10) compared with that in the Nivo+CT arm (22.2%, 2/9).

**Extended Data Fig. 9:**
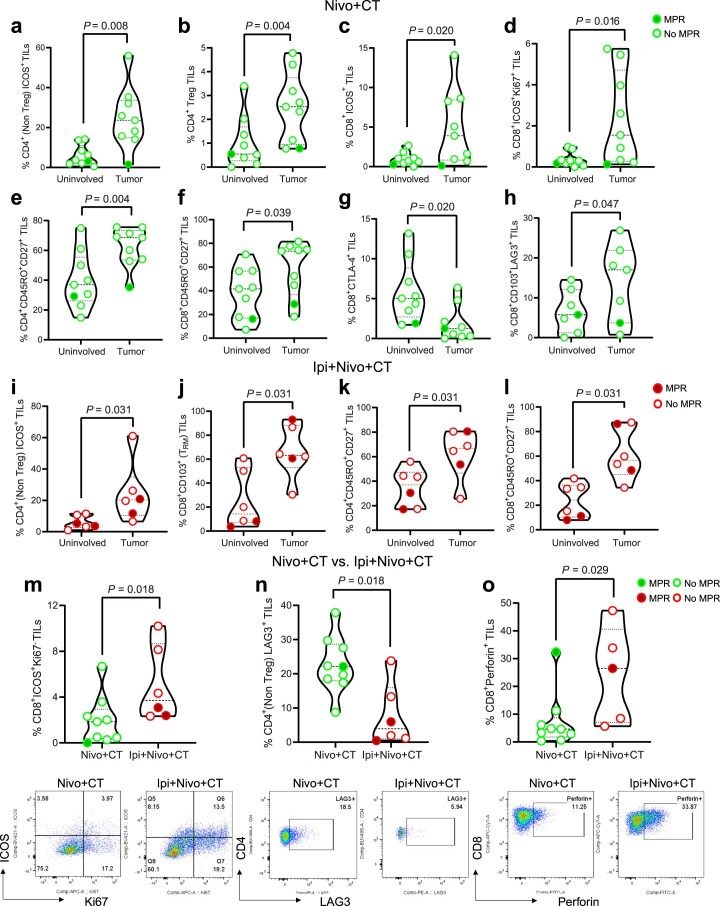
Immune infiltrates by flow cytometry analysis in resected uninvolved lungs and tumors treated with neoadjuvant Nivo+CT and Ipi+Nivo+CT. **a-h**, Flow cytometry in paired resected uninvolved lung (*n* = 9) and tumor tissues (*n* = 9) in Nivo+CT. Percentage of CD4+ (Non Treg) ICOS+ TILs (**a**), CD4+ Treg TILs (**b**), CD8+ ICOS+ TILs (**c**), CD8+ ICOS+ Ki67+ TILs (**d**), CD4+ CD45RO+ CD27+ TILs (**e**), CD8+ CD45RO+ CD27+ TILs (**f**), CD8+ CTLA-4+ TILs (**g**), CD8+ CD103+ LAG3+ TILs (**h**). **i-l**, Flow cytometry in paired resected uninvolved lung (*n* = 6) and tumor tissues (*n* = 6) in Ipi+Nivo+CT. Percentage of CD4+ (Non Treg) ICOS+ TILs (**i**), CD8+ CD103+ (tissue-resident memory T cells, T_RM_) TILs (**j**), CD4+ CD45RO+ CD27+ TILs (**k**), CD8+ CD45RO+ CD27+ TILs (**l**). **m**-**o**, Flow cytometry in resected tumor tissues after Nivo+CT (*n* = 9) and Ipi+Nivo+CT (*n* = 6 in **m** and **n**, *n* = 5 in **o**) treatments. Percentages (top panels) and representative gates (bottom panels) of CD8+ ICOS+ Ki67- TILs (**m**), CD4+ (Non Treg) LAG3+ TILs (**n**), CD8+ Perforin+ TILs (**o**). The green filled and empty circles depict data from MPR and no MPR, respectively, in Nivo+CT patients, and the red filled and empty circles depict data from MPR and no MPR, respectively, in Ipi+Nivo+CT patients. Data are presented as the median with minima, lower and upper quartiles, and maxima using violin plots. The dashed line shows the median value, and dotted lines show the lower quartile and upper quartile values of the range; the top and bottom of the violin plots indicate the maxima and minima. Two-sided *P* value is from Wilcoxon signed-rank test (**a**-**l**) and Wilcoxon rank-sum test (**m**-**o**). Experiments and gating related to presented results were conducted once. Subgating was performed only when more than 100 events were present in the parental gate. Nivo, nivolumab; Ipi, ipilimumab; CT, chemotherapy; TILs, tumor-infiltrating lymphocytes; MPR, major pathologic response. Source data is provided as Source Data file. Source data

To assess the impact of adding Ipi to a backbone of Nivo+CT on the tumor microenvironment, we used multiplex immunofluorescence (mIF) staining and flow cytometry of tissues pre-therapy and post-therapy. mIF analyses revealed significantly higher densities of CD3^+^CD8^+^ tumor-infiltrating T lymphocytes (TILs) in the Nivo+CT arm (*P* = 0.032; Extended Data Fig. 8d) and, to a greater extent, in the Ipi+Nivo+CT arm (*P* = 0.005; Extended Data Fig. 8d) after neoadjuvant therapy. Antigen-experienced and effector memory TIL densities increased in tumors after Nivo+CT compared with pre-therapy (Extended Data Fig. 8e,f), respectively, whereas the density of antigen-activated TILs was greater in tumors after Ipi+Nivo+CT compared with pre-therapy (Extended Data Fig. 8g). Examples of micrographs of mIF staining of pre-therapy and post-therapy TILs in tumor samples from both treatment arms are shown in Extended Data Fig. 8h–k. Flow cytometry analyses (subgating strategy is shown in Supplementary Fig. 7) revealed increased frequencies of activated (ICOS^+^) and proliferating (Ki67^+^) CD4^+^ and CD8^+^ TILs (Extended Data Fig. 9a–d), of CD4^+^ and CD8^+^ memory TILs (Extended Data Fig. 9e,f), reduced frequencies of CTLA-4^+^ immunosuppressive CD8^+^ TILs (Extended Data Fig. 9g) and increased percentages of CD8^+^CD103^+^LAG3^+^ TILs (Extended Data Fig. 9h) in tumors compared with uninvolved lungs treated with Nivo+CT. In tumors resected after Ipi+Nivo+CT, we observed an increase in the frequencies of CD4^+^-activated (ICOS^+^) and CD8^+^ tissue-resident memory TILs and memory T cells compared with uninvolved lungs (Extended Data Fig. 9i–l). Interestingly, we noted greater amounts of CD8^+^-activated and cytolytic TILs and reduced levels of CD4^+^LAG3^+^ TILs in tumors treated with Ipi+Nivo+CT compared with those treated with Nivo+CT (Extended Data Fig. 9m–o). Together these results corroborate the scRNA-seq findings and indicate greater immune activation, effector memory and cytotoxic function, along with attenuated immune suppression, in tumors treated with Ipi+Nivo+CT compared with Nivo+CT.

### Fecal microbiome

**Fig. 5:**
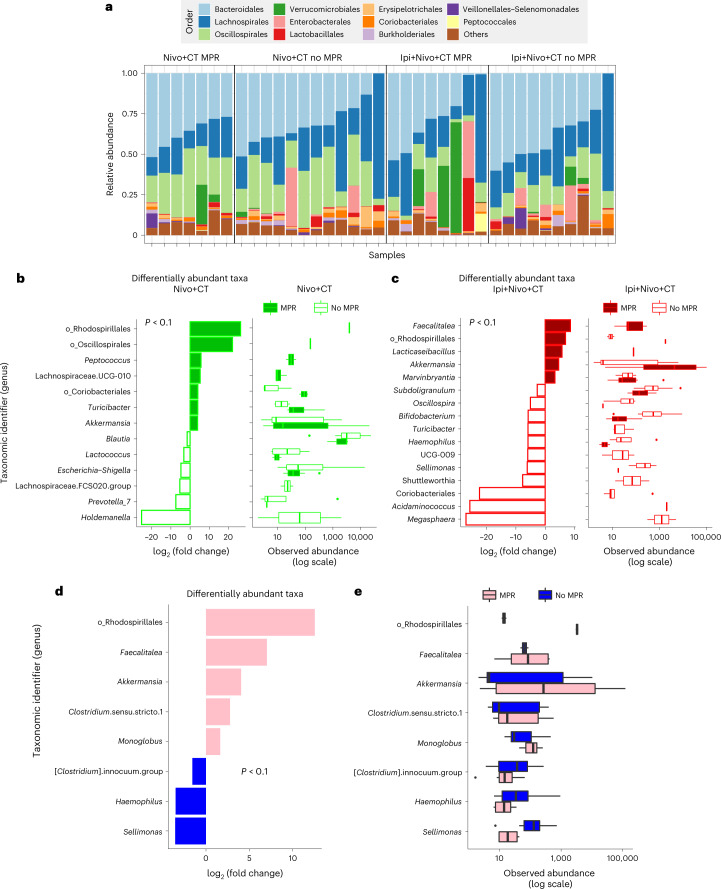
Association of fecal microbiome diversity and composition with responses to neoadjuvant Nivo+CT or Ipi+Nivo+CT. Fecal samples collected before Nivo+CT (*n* = 19) or Ipi+Nivo+CT (*n* = 18) treatments were characterized via 16S V4 RNA gene profiling. **a**, Fecal microbiome profiles of patient samples from the two treatment groups and MPR status are represented by compositional plots showing the relative abundance at the order level. **b**, Differentially abundant taxa (cutoff of *P* < 0.1, two-sided DESeq2 likelihood ratio test) aggregated at the genus level between MPR (*n* = 7) and no MPR (*n* = 12) in the Nivo+CT arm (left panel) and box-and-whisker plot (right panel) of DESeq2 normalized abundances. **c**, Differentially abundant taxa (cutoff of *P* < 0.1, two-sided DESeq2 likelihood ratio test) aggregated at the genus level between MPR (*n* = 8) and no MPR patie*n*ts (*n* = 10) in the Ipi+Nivo+CT arm (left panel) and box-and-whisker plot (right panel) of DESeq2 normalized abundances. **d**, Differentially abundant taxa (cutoff of *P* < 0.1, two-sided DESeq2 likelihood ratio test) aggregated at the genus level (or last known taxon) associated with the Nivo+CT and Ipi+Nivo+CT treatment responses—that is, MPR (*n* = 15) and no MPR (*n* = 22). **e**, Box-and-whisker plot of DESeq2 normalized abundances evaluating differentially abundant taxa associated with MPR status for the same taxa as shown in **d**. For box-and-whisker plots (**b**,**c**,**e**), the box includes data within first (Q1) and third (Q3) quartiles. The horizontal line represents the median. Length of whiskers represents minima (Q1 − [1.5 × IQR], where IQR means interquartile range) and maxima (Q3 + [1.5 × IQR]). Data points outside of whiskers are considered outliers. For differential abundance analyses (**b**,**c**,**d**), *P* values for each feature are provided in a source data file. Source data for **a–e** are provided in a source data file. Source data

**Extended Data Fig. 10:**
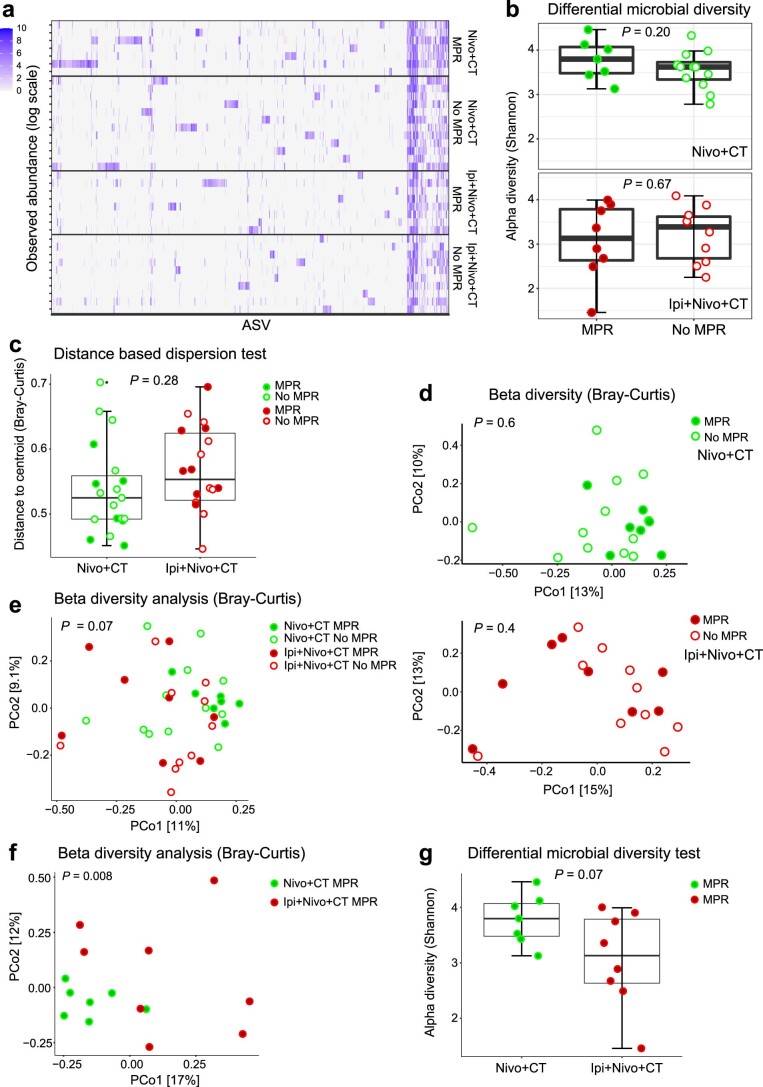
Association of MPR with fecal microbiome diversity and composition following neoadjuvant Nivo+CT or Ipi+Nivo+CT. Fecal samples collected before treatment were characterized via 16Sv4 RNA gene profiling in Nivo+CT (*n* = 19) or Ipi+Nivo+CT (*n* = 18) arms. **a**, Abundance (log-scale) of amplicon sequence variants (ASV) (in log-scale) observed in patients from both treatment groups with MPR and no MPR. **b**, Comparison of Shannon Index scores between patients with MPR and no MPR in each treatment arm (top, Nivo+CT; bottom: Ipi+Nivo+CT). **c**, Comparison of between-sample variance in between treatment arms using beta-dispersion test with Bray-Curtis dissimilarity. **d**, Principal coordinate analysis (PCoA) plots of Bray-Curtis distances comparing MPR versus no MPR (top, Nivo+CT; bottom: Ipi+Nivo+CT). **e**, Principal coordinate analysis (PCoA) plots of Bray-Curtis distances comparing samples from patients with MPR and no MPR from all treatment arms. **f**, Principal coordinate analysis (PCoA) plots of Bray-Curtis distances comparing MPR from both treatment arms (Nivo+CT *n* = 7, Ipi+Nivo+CT *n* = 8). **g**, Comparison of Shannon Index scores between MPR from both treatment groups (Nivo+CT *n* = 7, Ipi+Nivo+CT *n* = 8). For alpha diversity analyses (**b, g)** two-sided *P* value derived from ANOVA. For beta diversity analyses (**c–f**) two-sided *P* value derived from PERMANOVA with 999 permutations. For box and whisker plots (**b, c, g**), the box includes data within first (Q1) and third (Q3) quartiles. Horizontal line represents the median. Length of whiskers represent minima (Q1-[1.5xIQR]) and maxima (Q3+ 1.5xIQR]). Data points outside of whiskers are considered outliers. Nivo, nivolumab; Ipi, ipilimumab; CT, chemotherapy; MPR, major pathologic response. Source data is provided as Source Data file. Source data

The individual composition of fecal microbiomes of pre-treatment samples from the Nivo+CT (*n* = 19, 86%) and Ipi+Nivo+CT (*n* = 18, 82%) were dominated by bacteria from the Firmicutes and Bacteroidota phyla (Fig. 5a) and had similar distribution of identified taxa (Extended Data Fig. 10a). Differential abundance analyses revealed distinct signatures in patients with MPR compared to those without in each treatment arm (Fig. 5b,c) as well as in patients with MPR compared to those without in both arms combined (Fig. 5d,e). Bacteria of the order of Rhodospirillales and *Akkermansia* were consistently observed to be associated with MPR. In contrast, *Holdemanella* and *Megasphaera*, and *Haemophilus* and *Sellimonas*, were associated with lack of MPR in each arm and in the combined analysis, respectively. Additional analyses did not reveal differences in alpha-diversity (Extended Data Fig. 10b) or in beta-diversity (Extended Data Fig. 10c–e) in patients with MPR compared to those without. Interestingly, analyses of beta-diversity, but not alpha-diversity, revealed significant differences in patients with MPR in each group (Extended Data Fig. 10f,g). Together, our findings indicate that a favorable gut microbiome composition, including higher relative abundance of *Akkermansia* and reduced relative abundance of pro-invasive strains, was associated with response to therapy in our patient cohorts.

## Discussion

Until now, the pathologic and immunologic consequences of adding the CTLA-4 checkpoint inhibitor Ipi to neoadjuvant combined Nivo+CT for patients with resectable NSCLC have not been investigated. The NEOSTAR phase 2 platform trial evaluating neoadjuvant Nivo+CT and Ipi+Nivo+CT met its primary endpoint in both treatment arms, which exceeded the historical conservative MPR rate of approximately 15% produced by neoadjuvant CT. Neoadjuvant Nivo+CT produced an MPR rate of 32.1%, whereas Ipi+Nivo+CT resulted in an MPR rate of 50%. The addition of Ipi to Nivo+CT maintained an overall acceptable toxicity and allowed curative-intent surgery without adverse postoperative outcomes. Although the trial was not directly designed to compare both arms, our clinical and pathological findings of potential enhanced activity of Ipi+Nivo+CT are supported by our translational analyses demonstrating compositional changes consistent with marked tumor immune infiltration with an anti-tumor activity phenotype in tumors from the Ipi+Nivo+CT cohort compared with those treated with Nivo+CT.

MPR was selected as the primary outcome measure in our study owing to the low pCR rates achieved by CT alone in historical neoadjuvant trials and as demonstrated in the control CT arm of the CheckMate-816 study^3^. pCR has become a more relevant outcome measure only recently, since the report of 24.0% pCR rate in the CheckMate-816 trial^3^ and 36.8% pCR rate in the NADIM II trial^17^ in tumor *EGFR* and *ALK* wild-type patients. The results of our evaluation of Nivo+CT are overall consistent with the findings from CheckMate-816 (ref. ^3^), particularly when excluding tumors with known *EGFR/ALK* alterations. In CheckMate-816, Nivo+CT resulted in MPR and pCR rates of 36.9% and 24.0%, respectively, and a 12-month and 24-month EFS of 76.1% and 63.8%, respectively^3^. We found MPR and pCR rates of 41.2% and 23.5%, respectively, and a 12-month and 24-month EFS of 100% and 71%, respectively, in patients without known tumor *EGFR/ALK* alterations. The similarities between the two trials with respect to Nivo+CT treatment lend support for the NEOSTAR platform as a viable approach for evaluating new neoadjuvant therapies for resectable NSCLC. Furthermore, the extent of residual viable tumor (RVT) at surgery in patients treated with Ipi+Nivo+CT was a fraction of that found after Nivo+CT (median: 4.5% versus 50.5%). It is worth noting that 86% of patients with MPR in the Nivo+CT group and all patients with MPR in the Ipi+Nivo+CT group had <5% RVT in their tumor specimen. This observation is important in the context of the analysis from the CheckMate-816 trial, which identified 0–5% RVT as the most optimal cutoff associated with 90% 2-year EFS^18^. We also observed even greater tumor regression to Ipi+Nivo+CT in stage IIIA disease compared to Nivo+CT, which builds on the findings of the CheckMate-816 (ref. ^3^) and the phase 2 NADIM study^11,19^. These data suggest that more advanced tumors may require combination therapy, and addition of CTLA-4 blockade may achieve deeper pathologic responses in the stage III setting. We also observed less viable tumor cells after Ipi+Nivo+CT in tumors harboring *KRAS* and *TP53* alterations, consistent with prior results demonstrating improved outcomes to immunotherapy in these molecular subgroups^20–24^, similar to the exploratory analyses of the CheckMate-227 part 1 study, which revealed improved OS in patients with *KRAS*-mutant and *TP53*-mutant metastatic tumors treated with Ipi+Nivo compared with CT^25^.

Our scRNA-seq analysis demonstrated marked differences in the immune landscape between tumors and uninvolved normal tissues in both treatment arms. Tumors were characterized by increased fractions of CD4^+^ T_reg_ cells and Tfh, B cells and TAMs, consistent with previous reports on single-cell analyses of samples from treatment-naive patients with NSCLC^26,27^. Comparative scRNA-seq analysis unraveled conspicuous changes in the fractions of immune populations in tumors across both arms. Our findings of increased fraction of CD8^+^ TERM eff/TEM cells, a subset recently described in a pan-cancer T cell atlas^28^, in Ipi+Nivo+CT-treated tumors suggest their potential role in anti-tumor immune responses by the addition of Ipi. Our observations on increased abundance of B cells in Ipi+Nivo+CT relative to Nivo+CT and their inverse correlation with remaining viable tumor cells, along with our corroborative data on TLS genes by NanoString-based profiling, suggest an association of B lineage cells and TLS-associated genes, such as *CXCL13*, with immunotherapeutic response, as described previously^29–34^. B cells from Nivo+CT tumors showed increased expression of *CD24* that is reminiscent of CD24^hi^ B cell subsets that restrict T cell activation and cytokine production^35^. Bulk tumor immune profiling using the NanoString platform also showed elevated expression of *CD24* in tumors from the Nivo+CT arm, although it is conceivable that the overall greater viable tumor in this arm may account for increased expression of *CD24* (ref. ^26^). Also, our observation of increased *CXCL9*^+^ TAMs in tumors from patients treated with Ipi+Nivo+CT is in accordance with earlier studies suggesting a functional role for these macrophages in response to immune checkpoint therapy^36^. Overall, our single-cell sequencing analysis suggests that addition of Ipi to Nivo+CT favors a tumor ecosystem with overall enhanced tumor immune infiltrates and reduced immunosuppressive cell subsets and states. Additional support for this notion comes from the results of our tissue immune profiling with mIF and flow cytometry studies that revealed greater infiltration of antigen-activated (GZB^+^) CD8^+^ TILs, higher densities of activated (ICOS^+^) and cytolytic (perforin^+^) CD8^+^ TILs and reduced infiltration of LAG3^+^ immunosuppressive CD4^+^ TILs in tumors resected after Ipi+Nivo+CT treatment.

The tumor PD-L1 expression analysis was limited by the number of pre-therapy samples available for evaluation and the unexpected, particularly high incidence of tumors lacking PD-L1 expression on malignant cells in this dataset. This limited our ability to make firm conclusions regarding the association between this marker and therapeutic responses. Nevertheless, this cohort provided a unique opportunity to investigate the impact of treatment on responses in tumors lacking PD-L1 expression on cancer cells. Our findings suggest that Ipi may be particularly relevant to the treatment of PD-L1-negative tumors in which MPR was seen in 22.2% of patients treated with Nivo+CT and 40% of patients treated with Ipi+Nivo+CT. In patients with metastatic disease, the CheckMate-227 study (evaluating first-line Ipi+Nivo)^37^ and the CheckMate-9LA study (evaluating Ipi+Nivo+CT)^38^ suggested notable activity of Ipi in PD-L1-negative tumors. Benefits from neoadjuvant Nivo+CT (in ChekMate-816)^3^ and adjuvant atezolizumab after CT (in IMpower-010)^2^ were less in the PD-L1-negative subgroups, underscoring the need for improved perioperative strategies, possibly involving dual immunotherapy, for this patient subset.

The gut microbiome remains a strong tumor-extrinsic factor associated with anti-tumor response across various cancer histologies and treatment modalities. In this study, we observed different fecal microbiome structures and compositions between patients with MPR and those without MPR by treatment arm, suggesting a distinct association among treatment, response and gut microbiome composition. Notably, patients achieving MPR in both arms had fecal microbiomes enriched in *Akkermansia*, a mucin-degrading bacteria previously associated with responses to immunotherapy in NSCLC by our group^8^ and others^39^. Future larger studies will shed light on the mechanisms by which distinct microbial strains influence treatment outcomes and provide the foundation to evaluate the therapeutic benefit of additional microbiome modulation strategies in patients with cancers refractory to standard-of-care treatments.

In conclusion, our findings further support the role of neoadjuvant chemoimmunotherapy before NSCLC resection and expand on the standard-of-care neoadjuvant Nivo+CT by incorporating CTLA-4 blockade to this treatment regimen. The dual immune checkpoint therapy plus CT produces numerically higher MPR rates, is overall safe and tolerated, enhances anti-tumor immune activity and mitigates an immunosuppressive phenotype in exploratory analyses. The NEOSTAR platform trial design with surrogate endpoints and integrated multi-omic correlates enables the rapid assessment of promising therapeutic strategies and the identification of candidate targets to open new areas of translational investigation in the perioperative setting. The addition of CTLA-4 blockade to PD-(L)1 inhibition plus CT deserves further investigation for patients with resectable NSCLC.

## Methods

### Trial design, hypotheses and endpoints

This is a phase 2, open-label, single-institution, multi-arm study (NCT03158129) that, after completion of the first two randomized arms, evolved into a modular platform design^40^ with multiple, independent, single-arm studies expected to be analyzed and reported separately, with the goal to expedite the investigation of novel immunotherapy-based strategies in the neoadjuvant setting. The results of the first two randomized arms of the study have been reported^8^. New eligible patients were enrolled to the third arm (arm C) and treated with nivolumab plus platinum-based chemotherapy (Nivo+CT). Once the accrual of arm C was complete, eligible patients were enrolled to the fourth arm (arm D) and treated with ipilimumab plus nivolumab plus platinum-based chemotherapy (Ipi+Nivo+CT). The primary hypothesis to be tested was that, in patients with NSCLC amenable for surgical resection, induction therapy with Nivo+CT or Ipi+Nivo+CT will produce MPR rates of at least 40%, a target response rate that is superior to the one observed after induction platinum-based CT alone of 15% (as observed in MD Anderson historical controls^14^). The prespecified boundary for a treatment arm to be considered promising for further testing was ≥6 MPR in 21 evaluable patients. The secondary hypothesis to be tested was that Nivo+CT or Ipi+Nivo+CT would induce immune responses (as assessed by CD8^+^ TILs) and tumor shrinkage (as assessed by radiographic imaging) and improve survival outcomes (time to events including EFS and OS). The primary endpoint of the trial was MPR, defined as less than or equal to 10% viable tumor cells in the original resected tumor bed after neoadjuvant therapy on trial. Secondary endpoints included treatment toxicity, perioperative morbidity and mortality, quantification of CD8^+^ TILs in resected tumor tissues, objective response rate, pCR, completeness of surgical resection, time to events (including EFS and OS) and correlation of blood, tissue and stool biomarkers with efficacy. Exploratory endpoints included tissue-based, blood-based, stool-based and imaging-based biomarkers (Extended Data Fig. 1).

### Sample size justification and toxicity monitoring guidelines

Simon’s minimax two-stage design^41^ was applied to test the MPR rate for each of the treatment arms. We assumed a historical MPR rate of 15%^14^ under the null hypothesis versus an MPR rate of 40% under the alternative hypothesis. For each treatment arm, 15 patients were enrolled in the first stage. If only two or fewer of the 15 patients have experienced an MPR, enrollment to that treatment arm would be terminated, and the treatment would be considered inefficacious. Otherwise, with at least three MPRs, an additional six patients were enrolled to reach a total of 21 patients. At the end of each arm, if we observed six or more patients experiencing MPR, the treatment would be considered efficacious and inefficacious otherwise. Each arm has 90% power when the MPR rate is 40%. When the MPR rate is 15%, the probability of early termination is 60% with an average sample size of 17.4 and one-sided 10% type I error rate. From the above calculations, the study needs up to 21 evaluable patients in each arm. Assuming a non-evaluable rate of 5% (for example, patients drop out, become lost to follow-up or rescind consent due to non-treatment-related reasons before endpoints can be evaluated), we would need to enroll up to a total of 22 patients per arm. Enrolled patients were monitored for adverse events (AEs). AEs were treated as detailed in the protocol algorithm of toxicity management. We applied a Bayesian method to formally monitor the toxicity in the perioperative phase within each treatment arm^42^.

### Study oversight, ethical approval and ethical standards

Written informed consent was provided by all study participants or their legal representatives. The study was approved by The University of Texas MD Anderson Cancer Center’s institutional review board. Data were collected and analyzed by the investigators and interpreted by the authors. All authors approved and agreed to submit the final manuscript for publication. The authors vouch for the accuracy and completeness of the data and for the fidelity of the trial to the study protocol.

### Participants and neoadjuvant treatment

Male and female patients were screened, enrolled and treated at MD Anderson Cancer Center. The complete list of inclusion criteria is shown below:

1. Age >18 years.
2. Histologically or cytologically confirmed previously untreated NSCLC. If a diagnostic biopsy is available, a pre-treatment biopsy is not required. Patients with a suspected lung cancer are eligible, but pathology must be confirmed before initiating treatment on study. Neuroendocrine carcinomas are not eligible. Carcinomas with neuroendocrine differentiation are eligible.
3. Patients with stage IB ≥4 cm, IIA, IIB or IIIA disease (according to the American Joint Committee on Cancer 7th edition) are eligible for enrollment into arms C and D.
4. Patients with stage IIIA must not have more than one mediastinal LN station involved by tumor.
5. All patients must have LN evaluation of contralateral stations 2 and/or 4 to exclude N3 disease.
6. The patient must be a suitable candidate for surgery, in the opinion of the treating physician.
7. Signed and dated written informed consent must be provided by the patient before admission to the study in accordance with International Conference on Harmonisation Good Clinical Practice guidelines and to the local legislation.
8. Eastern Cooperative Oncology Group (ECOG) performance status (PS) score 0–1.
9. Patients must have organ and marrow function as defined below:

- Absolute neutrophil count ≥1.5 × 10^9^ per l
- Hemoglobin ≥8.0 g dl^−1^
- Platelets ≥100 × 10^9^ per l
- Total bilirubin ≤1.5× upper limit of normal (ULN) (except partients with Gilbert syndrome, who can have total bilirubin <3.0 mg dl^−1^)
- AST and ALT ≤3× ULN
- Creatinine ≤1.5× ULN or calculated creatinine clearance (Cockcroft–Gault formula for creatinine clearance calculation) ≥50 ml min^−1^ or 24-hour urine creatinine clearance ≥50ml min^−1^

The complete list of exclusion criteria is shown below:

1. Prior systemic therapy or radiation therapy for treatment of the current lung cancer.
2. Currently receiving cancer therapy (CT, radiation therapy, immunotherapy or biologic therapy) or investigational anti-cancer drug.
3. Pregnant or lactating female.

- Women of childbearing potential (WOCBP) must have a negative serum or urine pregnancy test (minimum sensitivity 25 IU L^−1^ or equivalent units of hCG) within 72 hours before the start of nivolumab.
- WOCBP is defined as any female who has experienced menarche and who has not undergone surgical sterilization (hysterectomy or bilateral oophorectomy) or who is not postmenopausal. Menopause is defined clinically as 12 months of amenorrhea in a woman over 45 years of age in the absence of other biological or physiological causes.

4. Unwillingness or inability to follow the procedures required in the protocol.
5. Patients with pre-existing sensorineural hearing impairment/loss or newly diagnosed as documented by an audiology assessment performed before study enrollment may not be eligible for cisplatin and may be dispositioned to carboplatin, as determined by the treating physician.
6. Patients with a history of severe hypersensitivity reaction to Taxotere and or polysorbate 80 must be excluded.
7. Any serious or uncontrolled medical disorder that, in the opinion of the investigator, may increase the risk associated with study participation or study drug administration, impair the ability of the patient to receive protocol therapy or interfere with the interpretation of study results. Prior malignancy active within the previous 2 years. Patients with locally curable cancers that have been apparently cured, such as basal or squamous cell skin cancer, superficial bladder cancer or carcinoma in situ of the prostate, cervix or breast with local control measures (surgery and radiation), are eligible.
8. Patients with active, known or suspected autoimmune disease. Patients with vitiligo, type I diabetes mellitus, residual hypothyroidism due to an autoimmune condition requiring only hormone replacement, psoriasis not requiring systemic treatment or conditions not expected to recur in the absence of an external trigger are permitted to enroll.
9. Patients with a condition requiring systemic treatment with either corticosteroids (>10 mg daily prednisone equivalents) or other immunosuppressive medications within 14 days of study drug administration. Inhaled or topical steroids and adrenal replacement doses >10 mg daily prednisone equivalents are permitted in the absence of active autoimmune disease.

- Patients are permitted to use topical, ocular, intra-articular, intranasal and inhalational corticosteroids (with minimal systemic absorption). Physiologic replacement doses of systemic corticosteroids are permitted, even if >10 mg per day prednisone equivalents. A brief course of corticosteroids for prophylaxis (for example, contrast dye allergy) or for treatment of non-autoimmune conditions (for example, delayed-type hypersensitivity reaction caused by contact allergen) is permitted.

10. Prior treatment with an anti-PD-1, anti-PD-L1 or anti-CTLA-4 antibody.
11. Known positive test for hepatitis B virus surface antigen or hepatitis C virus ribonucleic acid indicating acute or chronic infection.
12. Known history of testing positive for HIV or known AIDS.
13. History of severe hypersensitivity reaction to any monoclonal antibody and/or to study drug components.
14. Serious illness or concomitant non-oncological disease such as neurologic, psychiatric, infectious disease or laboratory abnormality that may increase the risk associated with study participation or study drug administration and, in the judgment of the investigator, would make the patient inappropriate for entry into the study.
15. Patients who are sexually active, with preserved reproductive capacity, and unwilling to use a medically acceptable method of contraception (for example, implants, injectables, combined oral contraceptives, some intrauterine devices or vasectomized partner for participating females and condoms for participating males) during and after the trial as detailed below:

- WOCBP should use an adequate method to avoid pregnancy for 23 weeks after the last dose of investigational drug(s).
  - Men who are sexually active with WOCBP must use any contraceptive method with a failure rate of less than 1% per year.
  - Men receiving nivolumab and who are sexually active with WOCBP will be instructed to adhere to contraception for a period of 31 weeks after the last dose of investigational product.
  - Women who are not of childbearing potential as well as azoospermic men do not require contraception.

16. Psychological, familial, sociological or geographical factors potentially hampering compliance with the study protocol and follow-up schedule.

Sex and/or gender was not considered in the trial design. Patient characteristics, including self-reported sex, are reported in Table 1. The participants were not compensated for their participation on the trial. The neoadjuvant treatment consisted of nivolumab 360 mg intravenously (IV) every 3 weeks (on day (D) 1, D22 and D43) plus cisplatin 75 mg per m^2^ (or carboplatin AUC 5 or 6) IV and docetaxel 75 mg per m^2^ IV administered every 3 weeks (on D1, D22 and D43), up to a maximum of three cycles for squamous histology NSCLC or nivolumab 360 mg IV every 3 weeks (on D1, D22 and D43) plus cisplatin 75 mg per m^2^ (or carboplatin AUC 5 or 6) IV and pemetrexed 500 mg per m^2^ IV administered every 3 weeks (on D1, D22 and D43), up to a maximum of three cycles for non-squamous histology NSCLC. For carcinomas with neuroendocrine features and/or differentiation, either regimen with nivolumab plus platinum and docetaxel or nivolumab plus platinum and pemetrexed were allowed based on the treating physician’s preference. In arm D, Ipi 1 mg per kg IV was administered on D1 of therapy only (cycle 1). Carboplatin was an option in arm D only.

### Pathologic assessment

Pathologic assessment consisted of gross and histopathologic examination of the lung resection specimens. After gross identification of the tumor or tumor bed, at least one section per centimeter of greatest tumor (bed) diameter was submitted for histopathological evaluation, as previously reported^14^. In cases in which no residual viable tumor was identified microscopically on initial representative sections and for tumors less than or equal to 3 cm in size, the entire tumor bed was submitted for review. In total, the tumor (bed) was submitted entirely in 38 cases. Histopathologically, the mean percentage of viable tumor cells, averaged across all reviewed tumor slides, was assessed for each patient as previously reported^14^. Tumors with less than or equal to 10% of viable tumor cells were considered to have undergone MPR, and tumors with 0% viable tumor were considered to have undergone pCR. After initial clinical reporting, pathologic responses were subsequently reviewed in a blinded manner by two pathologists experienced in the evaluation of tumor response after neoadjuvant therapy, and the average scores were used for final analysis as previously reported^8,43^. Mediastinal and peribronchial LNs were submitted and processed in a routine fashion for microscopic assessment and examined for metastatic disease. Pathologic staging was performed based on tumor and LN assessment of the resection specimens.

### Tumor molecular profiling

A next-generation sequencing (NGS)-based analysis for the detection of somatic variants of 146 cancer genes, including single-nucleotide variants (SNVs) of 134 genes and copy number gains of 47 genes, was performed on the DNA extracted from the available samples at the MD Anderson Cancer Center Clinical Laboratory Improvement Amendments (CLIA)-certified Molecular Diagnostics Laboratory (MDL). When possible, an in-house NGS-based analysis for the detection of targeted intergenic and intragenic fusions involving 51 cancer genes (RNA) was performed at the MD Anderson Cancer Center MDL. When possible, in-house fluorescence in situ hybridization assay (cytogenetics) was performed for *ALK*, *RET* and *ROS1* rearrangements and *MET* amplification. In some cases, tumor molecular profiling was obtained using in-house NGS-based analysis for the detection of SNVs in 70 genes, copy number gains in 19 genes and fusions in six genes performed on the plasma circulating cell-free DNA in our CLIA-certified MDL.

### Single-cell derivation and library preparation

Lung tissues were collected from seven patients with NSCLC who underwent surgical resection after neoadjuvant therapy with Nivo+CT (*n* = 2 patients) or Ipi+Nivo+CT (*n* = 5 patients). Tumor and matched uninvolved normal lung tissues from the seven patients, as well as a LN sample from one of the patients, were freshly obtained under clinical trial protocol and approved by the MD Anderson institutional review board. Tissues were collected in ice-cold DMEM medium supplemented with 2% FBS and immediately minced and enzymatically digested in DMEM containing 0.16 mg/ml of DNase I (9003-98-9, Worthington Biochemical) and 328 U/ml of Liberase (5401020001, Roche) for 30 minutes at 37 °C. Lysate was filtered and washed, after which red blood cells were eliminated using red blood lysis buffer (A1049201, Gibco). Total cells were cryopreserved in FBS with 10% DMSO and stored in the vapor phase of a liquid nitrogen tank until further processing. At the time of scRNA-seq library preparation, cryopreserved cells were thawed and washed twice with pre-warmed 2% FBS in PBS and then stained with viability marker (SYTOX Blue, S34857, Thermo Fisher Scientific) at room temperature in the dark. Viable singlets from each sample were sorted into 2% FBS in PBS using a BD FACSAria cell sorter. Sorted cells were maintained on ice before being washed, filtered, manually counted using a hemocytometer and trypan blue (T8154, Sigma-Aldrich) exclusion and resuspended in 2% FBS in PBS at 1,000 cells per microliter. Viable single cells were loaded on 10x Chromium microfluidic chips, and single-cell gene expression libraries were generated as previously described^26^ and according to the manufacturer’s standard protocols (Chromium 5′ Next GEM Single Cell Kit version 1.0, 1000006, 10x Genomics) and targeting 1,300-10,000 cells per sample. Single cells loaded onto Chromium Next GEM Chips A (2000167, 10x Genomics) were partitioned into nanoliter-scale gel beads-in-emulsion (GEMs) using Chromium Next GEM Single Cell 5′ Gel Bead Kit version 1.0 (1000003, 10x Genomics). Recovered barcoded GEMs were broken, pooled and underwent magnetic bead clean-up (Dynabeads MyOne Silane, 37002D, Thermo Fisher Scientific) to construct single-cell gene expression libraries using the Chromium Next GEM Single Cell 5′ Library kit (1000002, 10x Genomics) according to the manufacturer’s protocol. Next, 10x-barcoded full-length cDNA was amplified by PCR and analyzed using Bioanalyzer High Sensitivity DNA Kit (5067-4626, Agilent). Up to 50 ng of cDNA was subjected to enzymatic fragmentation and size selection to optimize the cDNA amplicon size before 5′ gene expression library construction. Finally, Illumina-ready barcoded gene expression libraries were generated after a round of end-repair, A-tailing, adaptor ligation and sample index PCR using Chromium i7 Multiplex Kit (120262, 10x Genomics). Library quality and yield were measured using Bioanalyzer High Sensitivity DNA (5067-4626, Agilent) and Qubit dsDNA High Sensitivity Assay (Q32854, Thermo Fisher Scientific) kits. Pooling of indexed libraries was done after adjustment of the ratio of the targeted cells per library as well as individual library concentration. Library pools were then denatured and diluted as recommended for sequencing on the Illumina NovaSeq 6000 platform. After quality control assessment, libraries were pooled and sequenced at a target depth of ~50,000 reads per cell on the Illumina NovaSeq 6000 platform.

### scRNA-seq analysis

scRNA-seq analysis was performed using available computational framework. The raw reads were aligned to human reference genome GRh38 (hg38) and processed by 10x Genomics Cell Ranger version 3.1.0 to generate the unique molecular identifier (UMI) count data matrix. The UMI data matrix was processed using the Seurat package (version 3)^44^, with the following workflow. (1) Data filtering: The UMI data matrix was filtered to remove genes that were not expressed in any cells as well as cells with fewer than 300 expressed genes or more than 10% of total UMI count of mitochondrial genes. (2) Data normalization and integration: Filtered UMI data matrices from different data batches were normalized, scaled, batch corrected and integrated using the data integration workflow in Seurat version 3, with the integration anchor features set to all genes in filtered datasets^44^. (3) Data reduction and visualization: Principal component analysis was performed using highly variable genes identified using the Seurat version 3 ‘VariableFeatures’ function. The top-ranked principal components that covered 80% of the total variance were selected and transformed into uniform manifold approximation and projection (UMAP) components for visualization. (4) Unsupervised clustering: Cell clusters were identified using the Seurat ‘FindClusters’ function, with resolution value manually adjusted to find the best separation. (5) Cluster annotation: The marker genes for each cluster were identified using the Seurat version 3 ‘FindClusterMarkers’ function. These markers genes, combined with gene markers for known cell types, such as immune cells and epithelial cells, were used to identify the major cell lineages of each cluster. Each cell lineage was further clustered to identify sublineages if needed. During these processes, additional doublets were identified and removed from the clusters. These clustering/identification processes were performed iteratively until all cell populations were annotated. (6) Differential analysis: For each cell population, we identified the differentially expressed genes between sample types (tumor versus uninvolved) and treatment group (Nivo+CT versus Ipi+Nivo+CT), using the Wilcoxon rank-sum test, with statistical cutoff set to false discovery rate less than 0.05 and log_2_ fold change greater than 1. Cell proportions between sample types (tumor versus uninvolved) and treatment group (Nivo+CT versus Ipi+Nivo+CT) were compared using two-sided proportion test. All statistical analyses were performed in R version 4.0.1.

### NanoString analysis

Surgically resected post-treatment formalin-fixed paraffin-embedded (FFPE) tissue samples from 19 Nivo+CT-treated and 19 Ipi+Nivo+CT-treated patients were cut into 4-µm-thick sections and shipped to the Immunotherapy Platform at our institution for NanoString analysis. The analysis was performed as per the umbrella protocol PA13-0291. Tissue sections were dewaxed using deparaffinization solution (Qiagen), and total RNA was extracted using the RecoverALL Total Nucleic Acid Isolation Kit (Ambion) according to the manufacturer’s instructions. RNA quality and quantity were assessed using the NanoDrop spectrometer (NanoDrop ND-1000, Thermo Fisher Scientific). For the assay, 100 ng of RNA was used to detect immune gene expression using the nCounter PanCancer Immune Profiling panel along with custom CodeSet. nCounter Digital Analyzer was used to tabulate the counts of the reporter probes, and, for further analysis, raw data output was imported into nSolver analysis software (version 4.0.70) (http://www.nanostring.com/products/nSolver). Normalization, cell type and differential gene expression analyses were performed using the nSolver Advanced data analysis package (version 2.0.134). The TLS signature score shown in Extended Data Fig. 7 is derived from the median expression of the following genes: *CCL19*, *CCL21*, *CXCL13*, *CCR7*, *SELL*, *LAMP3*, *CXCR4*, *CD86* and *BCL6* (ref. ^30^). The TLS signature score shown in Supplementary Fig. 6 is derived from the median expression of the following genes: *CD79A*, *MS4A1*, *LAMP3* and *POU2AF1* (ref. ^32^). Data were collected using Microsoft Excel version 2016 and plotted using GraphPad Prism version 9.0.0.

### Multiparameter flow cytometry

Fresh uninvolved tumor-adjacent normal lung and tumor tissues collected at surgery were disaggregated using the BD Medimachine System (BD Biosciences) to make a single-cell suspension for flow cytometry staining. Disaggregated cells were Fc-blocked using 5% goat serum (G9023, Sigma-Aldrich) for 30 minutes on ice. Surface staining was performed in 1× DPBS with 1% BSA (A8577, Sigma-Aldrich) for 30 minutes on ice using fluorochrome-conjugated monoclonal antibodies against CD45 (BUV395, clone HI30, 563792, BD Biosciences, 5 µl per sample), CD3 (PerCP-Cy5.5, clone SK7, 340949, BD Biosciences, 10 µl per sample), CD8 (AF700, clone RPA-T8, 557945, BD Biosciences, 5 µl per sample), CD4 (BUV496, clone SK3, 612936, BD Biosciences, 5 µl per sample), PD-1 (SB645, clone MIH4, 64-9969-42, eBioscience, 4 µl per sample), TIM3 (BV605, clone F38-2E2, 345018, BioLegend, 4 µl per sample), CD103 (BV711, clone Ber-Act8, 563162, BD Biosciences, 5 µl per sample), CTLA-4 (BV786, clone BNI3, 563931, BD Biosciences, 3 µl per sample), GITR (AF488, clone eBioAITR, 53-5875-42, eBioscience, 5 µl per sample), LAG3 (PE, clone 3DS223H, 2-2239-42, eBioscience, 5 µl per sample), CD56 (PE-Cy7, clone B159, 557747, BD Biosciences, 5 µl per sample), ICOS (BV421, clone C398.A4, 313524, BioLegend, 5 µl per sample) and CD25 (APCFire/750, clone BC96, 302642, BioLegend, 5 µl per sample). After surface staining, cells were fixed and permeabilized using eBioscience Foxp3/Transcription Factor Staining Buffer Set (00-5523-00, Thermo Fisher Scientific) according to the manufacturer’s instructions and stained using FOXP3 (PE-eFluor610, clone PCH101, 61-4776-42, eBioscience, 5 µl per sample) and Ki67 (APC, clone 20Raj1, 17-5699-42, eBioscience, 5 µl per sample) anti-human antibodies. For the memory panel, Fc-blocked cells were surface stained for 30 minutes on ice using monoclonal antibodies against CD27 (FITC, clone M-T271, 555440, BD Biosciences, 20 µl per sample), CCR7 (PerCP-Cy5.5, clone 150503, 561144, BD Biosciences, 5 µl per sample), CD45RA (V450, clone HI100, 560362, BD Bioscience, 5 µl per sample), CD3 (APC, clone UCHT1, 555335, BD Biosciences, 20 µl per sample), CD4 (BUV496, clone SK3, 612936, BD Biosciences, 5 µl per sample), CD8 (AF700, clone RPA-T8, 557945, BD Biosciences, 5 µl per sample), CD45RO (APC-H7, clone UCHL1, 561137, BD Biosciences, 5 µl per sample), BTLA (PE, clone J168-540, 558485, BD Biosciences, 5 µl per sample) and CD28 (PE-Cy7, clone CD28.2, 560684, BD Biosciences, 5 µl per sample). For the functional panel, Fc-blocked cells were surface stained for 30 minutes on ice using monoclonal antibodies against PD-1 (PerCP-Cy5.5, clone EH12, 329914, BioLegend, 5 µl per sample), TIM3 (APC, clone F38-2E2, 17-3109-42, eBioscience, 5 µl per sample), CD8 (APC-Cy7, clone RPA-T8, 557760, BD Biosciences, 3 µl per sample) and CD3 (PE-Cy7, clone UCHT1, 563423 BD Biosciences, 5 µl per sample). After staining, cells were fixed using the BD Fix/Perm buffer solution from the Fixation/Permeabilization Kit (554714, BD Biosciences) by incubating them for 20 minutes in the dark at room temperatures. Cells were then washed and stained with monoclonal antibodies against perforin (FITC, clone DG9, 11-9994-42, eBiosciences, 5 µl per sample), granzyme B (V450, clone GB11, 561151, BD Biosciences, 5 µl per sample) and IFNγ (PE, clone B27, 559327, BD Biosciences, 10 µl per sample) anti-human antibodies using the BD Perm Buffer I solution that was diluted with water according to the manufacturer’s instructions. Cells were stained for 30 minutes on ice for intracellular markers. Dead cells were stained using LIVE/DEAD Fixable Yellow Dead Cell Stain dye (L-34968, Life Technologies, 1 µl per sample) and excluded from the analysis. Data were acquired using the BD Fortessa X20 or Canto II (BD Bioscience) with BD FACSDiva software version 8.0.1 and analyzed using FlowJo software version 10.5.3 (Tree Star). Experiments and gating related to the presented results were conducted once. Detailed information on flow cytometry antibody panels is provided in Supplementary Table 12. The associated gating strategies are shown in Supplementary Fig. 7. The results were graphed using Microsoft Excel version 2016 and GraphPad Prism version 9.00.

### mIF staining and analysis

For mIF staining, reagents were validated, and similar methods to those previously described were applied^45^. Using an automated staining system (BOND-RX, Leica Microsystems), 4-μm-thick FFPE tumor sections were stained for two panels containing antibodies against: panel 1: cytokeratin (clone AE1/ AE3, M351501-2, dilution 1:300, Dako), CD3 (IS503, dilution 1:100, Dako), CD8 (clone C8/144B, MS-457-S, dilution 1:300, Thermo Fisher Scientific), CD68 (clone PG-M1, M0875, dilution 1:450, Dako), PD-1 (clone EPR4877-2, ab137132, dilution 1:250, Abcam) and PD-L1 (clone E1L3N, 13684S, dilution 1:3,000, Cell Signaling Technology); panel 2: cytokeratin (clone AE1/AE3, M351501-2, dilution 1:300, Dako), CD3 (IS503, dilution 1:100, Dako), CD8 (clone C8/144B, MS-457-S, dilution 1:300, Thermo Fisher Scientific), CD45RO (clone UCHL1, PA0146, Cell Signaling Technology), granzyme B (clone 11F1, PA0291, Cell Signaling Technology) and FOXP3 (clone D2W8E, 98377S, Cell Signaling Technology). All the markers were stained in sequence using their respective fluorophore contained in the Opal 7 kit (NEL797001KT, Akoya Biosciences/PerkinElmer). The stained slides were scanned using the multispectral microscope, Vectra version 3.0.3 imaging system (Akoya Biosciences/PerkinElmer), under fluorescence conditions in low (×10) magnification^46^. After the scanning phase in low magnification, a pathologist selected around five regions of interest (ROIs; each ROI: 0.3345 mm^2^) per sample to cover around 1.65 mm^2^ of tumor tissue using the Phenochart version 1.0.9 viewer (Akoya Biosciences/PerkinElmer). The ROIs were analyzed by a pathologist using InForm version 2.8.2 image analysis software (Akoya Biosciences/PerkinElmer). In panel 1, marker co-localization was used to identify malignant cells expressing (AE1/AE3^+^), malignant cells expressing PD-L1 (AE1/AE3^+^PD-L1^+^), T cell population expressing (CD3^+^), cytotoxic T cells (CD3^+^CD8^+^), antigen-experienced T cells (CD3^+^PD-1^+^), cytotoxic antigen-experienced T cells (CD3^+^CD8^+^PD-1^+^), T cells PD-L1^+^ (CD3^+^PD-L1^+^), cytotoxic T cells PD-L1^+^ (CD3^+^CD8^+^PD-L1^+^), cytotoxic T cells antigen-experienced expressing PD-L1^+^ (CD3^+^CD8^+^PD-1^+^PD-L1^+^), macrophages (CD68^+^) and macrophages expressing PD-L1 (CD68^+^PD-L1^+^). In panel 2, the positive expression of CD3 protein surface was used to identify T cells (CD3^+^), and the co-localization of more than one protein surface marker was used to identify cytotoxic T cells (CD3^+^CD8^+^), cytotoxic activated T cells (CD3^+^CD8^+^granzyme B^+^), memory T cells (CD3^+^CD45RO^+^), effector/memory cytotoxic T cells (CD3^+^CD8^+^CD45RO^+^), T_reg_ cells ((CD3^+^FoxP3^+^)−(CD3^+^CD8^+^FOXP3^+^)) and memory/T_reg_ cells (CD3^+^CD45RO^+^FoxP3^+^). Densities of each co-localized cell population were quantified as the average, and the final data were expressed as number of cells per mm^2^ in two compartments: tumor nests and tumor stroma^47^. Malignant cells and macrophages expressing PD-L1 were also expressed as percentages. All the data were consolidated using R Studio version 3.5.3 (Phenopter version 0.2.2 packet, Akoya Biosciences/PerkinElmer) and SAS version 7.1 Enterprise. Experiments and scorings related to the presented micrographs were conducted once. The data were collected using Microsoft Excel version 2016 and plotted using GraphPad Prism version 9.00.

### IHC of PD-L1 and analysis

We used FFPE tumor tissues to perform single chromogenic IHC analysis for PD-L1 (clone 28-8, ab205921, dilution 1:100, Abcam) using a Leica BOND-MAX autostainer system (Leica Biosystems). The optimal conditions were previously validated^48^ and are described here in brief. We used the automated standard Leica protocol in which tissue sections were first deparaffinized and rehydrated. Antigen retrieval was performed with BOND Solution 2 (Leica Biosystems, equivalent to ethylenediaminetetraacetic acid, pH 9.0) for 20 minutes. The primary antibody was incubated for 15 minutes at room temperature and detected using the BOND Polymer Refine Detection Kit (Leica Biosystems) with 3,3′-diaminobenzidine as the chromogen. Finally, the slides were counterstained with hematoxylin, dehydrated and cover-slipped. PD-L1 stained slides were scored by standard microscopy following the recommendations of the International Association for the Study of Lung Cancer guidelines^49^. Two pathologists evaluated PD-L1 expression in the membrane of viable malignant cells, and the results are reported as percentage of malignant cells with any positive membrane staining (tumor proportion score). Experiments and scorings related to the presented micrographs were conducted once. The data were collected using Microsoft Excel version 2016 and plotted using and GraphPad Prism version 9.00.

### Fecal microbiome specimen processing and analyses

Thirty-seven fecal samples collected before treatment were characterized via 16S V4 ribosomal RNA gene profiling. Fecal samples were processed as described previously^8^. In brief, fecal DNA was extracted using the QIAamp DNA Stool Kit (Qiagen), including a bead-beating lysis step. The V4 region of the bacterial 16S rRNA gene was amplified and sequenced on the Illumina MiSeq platform using the 2 × 250-bp paired-end protocol yielding paired-end reads with near-complete overlap. Raw FASTQ files were processed using DADA2 (1.18)^50^ to generate amplicon sequence variants and taxonomies assigned with SILVA version 138 (ref. ^51^). The resulting amplicon sequence variant table and taxonomies were used to compute alpha-diversity and beta-diversity metrics as well as taxonomic relative abundances.

### Statistical methods

At the completion of the first stage (15 patients) of the Simon’s minimax two-stage design in each study arm, the number of MPR was evaluated. The study would proceed to the second stage if the number was greater than the critical value of two. In the Nivo+CT arm, six MPRs were achieved in the first stage, and, thus, the study proceeded to the second stage. In the Ipi+Nivo+CT arm, seven MPRs were achieved in the first stage, and, thus, the study proceeded to the second stage. As the primary analysis, a uniformly minimum variance unbiased estimator (UMVUE) of the MPR rate was obtained using the approach proposed by Jung and Kim^52^ within each study arm. A *P* value for the statistical test against the assumed historical control of 15% and the corresponding 80% two-sided CI was calculated using the method developed by Koyama and Chen^53^ to adjust for the Simon’s two-stage design’s adaptiveness. TRAEs are summarized with frequencies and percentages. Subsequent analyses comparing the two treatments arms are considered as exploratory in nature. For correlative analyses, the normality was checked before the Wilcoxon rank-sum test or *t*-test was used to compare continuous variables between two independent groups. The Wilcoxon signed-rank test was used for comparison of paired data. Exact tests were performed where applicable. A univariate logistic regression model was used to assess the association between characteristics and MPR in combined arms and between treatment arm and MPR in characteristics subgroups. Time-to-event analyses, including EFS and OS, were performed. EFS was defined as the time from treatment initiation to any progression of primary lung cancer precluding planned surgery, any progression or recurrence (as assessed by imaging and/or histopathologically) of primary lung cancer after surgery, any progression of primary lung cancer in patients without surgery or death from all causes or to the time of last imaging. OS was defined as the time from treatment initiation to the time of death from all causes or to the time of last follow-up. Obituaries were cross-referenced for any unreported patient deaths. The landmark method^54^ was applied to conduct EFS analysis by pathologic response assessed at surgery in the primary lung cancer. In the landmark analysis, EFS was defined as the time from surgery to any recurrence (as assessed by imaging and/or histopathologically) caused by primary lung cancer or death from all causes or to the time of last imaging. The distributions of EFS and OS were estimated by the Kaplan–Meier method^55^. The log-rank test^56^ was performed to test the difference in survival between groups. A two-sided *P* value of 0.05 was considered significant. All analyses were performed in SAS version 9.4 and R version 4.1.2. For microbiome analyses, beta-diversity distances were calculated using Bray–Curtis dissimilarity and represented on principal coordinate analysis plots using the top two principal components. PERMANOVA^57^ analyses (with 999 permutations) and beta-dispersion tests were used to compare microbiota diversity and dispersion between groups. Differential abundance analysis of identified taxonomies was performed with DESeq2 (ref. ^58^) and implemented in R^59^ along with additional statistical analyses and illustrations using ggplot2 (ref. ^60^).

### Reporting summary

Further information on research design is available in the Nature Portfolio Reporting Summary linked to this article.

## Online content

Any methods, additional references, Nature Portfolio reporting summaries, source data, extended data, supplementary information, acknowledgements, peer review information; details of author contributions and competing interests; and statements of data and code availability are available at 10.1038/s41591-022-02189-0.

## Supplementary information

Supplementary Information Supplementary Figs. 1–7.

Reporting Summary

Supplementary Table 1 Tumor molecular alterations in both treatment arms. Splice, an alteration of genomic DNA adjacent to the intron/exon splice border, which could have an effect on RNA splicing, has been identified. *Molecular tests performed at outside institution.

Supplementary Table 2 Pathologic responses in the ITT population in both treatment arms. Two Ipi+Nivo+CT patients were not resected due to death from SARS-CoV-2 infection-related complications (non-treatment related) in one patient and complexity of surgical resection and its associated risks in the second patient. *MPR rate was obtained from a UMVUE. VT, viable tumor; NE, not evaluable.

Supplementary Table 3 Pathologic responses in all resected patients in both treatment arms. VT, viable tumor.

Supplementary Table 4 Univariate logistic regression model to assess the association between clinical characteristics and MPR in combined treatment arms. OR, odds ratio; NOS, not otherwise specified; Squamous, squamous cell carcinoma. Non-squamous includes adenocarcinoma, carcinoma with neuroendocrine features, NOS NSCLC, sarcomatoid carcinoma and large cell carcinoma.

Supplementary Table 5 Pathologic responses in the ITT population without known tumor *EGFR* and *ALK* alterations in both treatment arms. VT, viable tumor; NE, not evaluable.

Supplementary Table 6 TRAEs in both treatment arms. eGFR, estimated glomerular filtration rate; BUN, blood urea nitrogen.

Supplementary Table 7 SAEs by grade in both treatment arms. *One patient died of SARS-CoV-2 infection-related complications (non-treatment related).

Supplementary Table 8 Neoadjuvant treatment, pathologic response attributes and molecular alterations of patients with scRNA-seq of treated tumor and uninvolved (normal) lung tissues.

Supplementary Table 9 Immune gene signature scores by NanoString in *EGFR* mutant/*ALK* rearranged versus *EGFR* wt/*ALK* wt tumors. Gene expression analysis was performed by NanoString technology on post-treatment tumor tissue samples from patients treated with Nivo+CT (*n* = 14) and Ipi+Nivo+CT (*n* = 13) and with available tumor molecular profiling. TLS^*^ signature score is derived from the median expression of *CCL19*, *CCL21*, *CXCL13*, *CCR7*, *SELL*, *LAMP3*, *CXCR4*, *CD86* and *BCL6* genes. TLS^#^ signature score is derived from the median expression of *CD79A*, *MS4A1*, *LAMP3* and *POU2AF1* genes. Two-sided *P* values are from Wilcoxon rank-sum test for the analysis of CD45 cells, T cells, CD8 T cells, cytotoxic cells and macrophage median scores. Two-sided *P* values are from unpaired *t*-test for the analysis of NK cells, B cells, TLS^*^ and TLS^#^ mean scores. wt, wild type.

Supplementary Table 10 Immune gene signature scores by NanoString in *KRAS* mutant versus *KRAS* wt tumors. Gene expression analysis was performed by NanoString technology on post-treatment tumor tissue samples from patients treated with Nivo+CT (*n* = 14) and Ipi+Nivo+CT (*n* = 13) and with available tumor molecular profiling. TLS^*^ signature score is derived from the median expression of *CCL19*, *CCL21*, *CXCL13*, *CCR7*, *SELL*, *LAMP3*, *CXCR4*, *CD86* and *BCL6* genes. TLS^#^ signature score is derived from the median expression of *CD79A*, *MS4A1*, *LAMP3* and *POU2AF1* genes. Two-sided *P* values are from Wilcoxon rank-sum test. wt, wild type.

Supplementary Table 11 Immune gene signature scores by NanoString in *TP53* altered versus *TP53* wt tumors. Gene expression analysis was performed by NanoString technology on post-treatment tumor tissue samples from patients treated with Nivo+CT (*n* = 14) and Ipi+Nivo+CT (*n* = 13) and with available tumor molecular profiling. TLS^*^ signature score is derived from the median expression of *CCL19*, *CCL21*, *CXCL13*, *CCR7*, *SELL*, *LAMP3*, *CXCR4*, *CD86* and *BCL6* genes. TLS^#^ signature score is derived from the median expression of *CD79A*, *MS4A1*, *LAMP3* and *POU2AF1* genes. Two-sided *P* values are from Wilcoxon rank-sum test for the analysis of CD45 cells, T cells and cytotoxic cells median scores. Two-sided *P* values are from unpaired *t*-test for the analysis of CD8 T cells, NK cells, B cells, TLS^*^, macrophages and TLS^#^ mean scores. wt, wild type.

Supplementary Table 12 Information on flow cytometry antibody panels.

Source Data for Supplementary Fig. 6 Processed data and annotations.

## Data Availability

De-identified scRNA-seq raw data reported in this paper have been deposited in the European Genome-phenome Archive (EGA) with accession number EGAS00001006728. Access to this dataset is controlled by the institutional Data Access Committee in compliance with National Institutes of Health policy for Data Management and Sharing and in accordance with an alliance agreement between MD Anderson Cancer Center and Bristol Myers Squibb. Access to this dataset will be granted upon review and acceptance of academic requests. Further information about the EGA can be found at https://egaarchive.org. The raw reads were aligned to human reference genome GRCh38 (hg38). The 16S fecal microbiome sequencing data have been deposited in the National Center for Biotechnology Information Sequence Read Archive (SRA) under SRA BioProject ID PRJNA665109. Taxonomies were assigned with SILVA database version 138 (https://www.arb-silva.de). Source data are provided with this paper.

## Source data

Source Data Fig. 5 Processed data and annotations.
Source Data Extended Data Fig. 7 Processed data and annotations.
Source Data Extended Data Fig. 8 Processed data and annotations.
Source Data Extended Data Fig. 9 Processed data and annotations.
Source Data Extended Data Fig. 10 Processed data and annotations.

## Code availability

Codes used for scRNA-seq analysis are available from https://github.com/MD-Anderson-Bioinformatics/Neoimmuno.
