# Supplementary material for: Neoadjuvant chemotherapy plus nivolumab with or without ipilimumab in operable non-small cell lung cancer: the phase 2 platform NEOSTAR trial
Source: Nat Med. 2023 Mar 16;29(3):593–604. doi: 10.1038/s41591-022-02189-0 (PMC10033402; doi:10.1038/s41591-022-02189-0)
Supplement: Supplementary file 1 — Supplementary Figs. 1–7. [file 41591_2022_2189_MOESM1_ESM.pdf]

nature medicine

Article https://doi.org/10.1038/s41591-022-02189-0

Neoadjuvant chemotherapy plus nivolumab with or without ipilimumab in operable non-small cell lung cancer: the phase 2 platform NEOSTAR trial

In the format provided by the authors and unedited

Supplementary Information File

Table of Content

Supplementary Figure 1……………………………………………………………..………Page 2

Supplementary Figure 1 Legend……………………………………………………...……Page 3

Supplementary Figure 2……………………………………………………………..………Page 4

Supplementary Figure 2 Legend………………………………………………………..…Page 5

Supplementary Figure 3…………………………………………………………….…..……Page 6

Supplementary Figure 4…………………………………………………………….…..……Page 8

Supplementary Figure 3 Legend………………………………………………..…..……Page 7

Supplementary Figure 4 Legend……………………………………………………...……Page 9

Supplementary Figure 5…………………………………………………………………...…Page 10

Supplementary Figure 5 Legend……………………………………………………….…Page 11

Supplementary Figure 6……………………………………………………………..…...…Page 12

Supplementary Figure 6 Legend………………………………………………………...…Page 13

Supplementary Figure 7……………………………………………………………..…...…Page 14

Supplementary Figure 7 Legend………………………………………………………...…Page 15

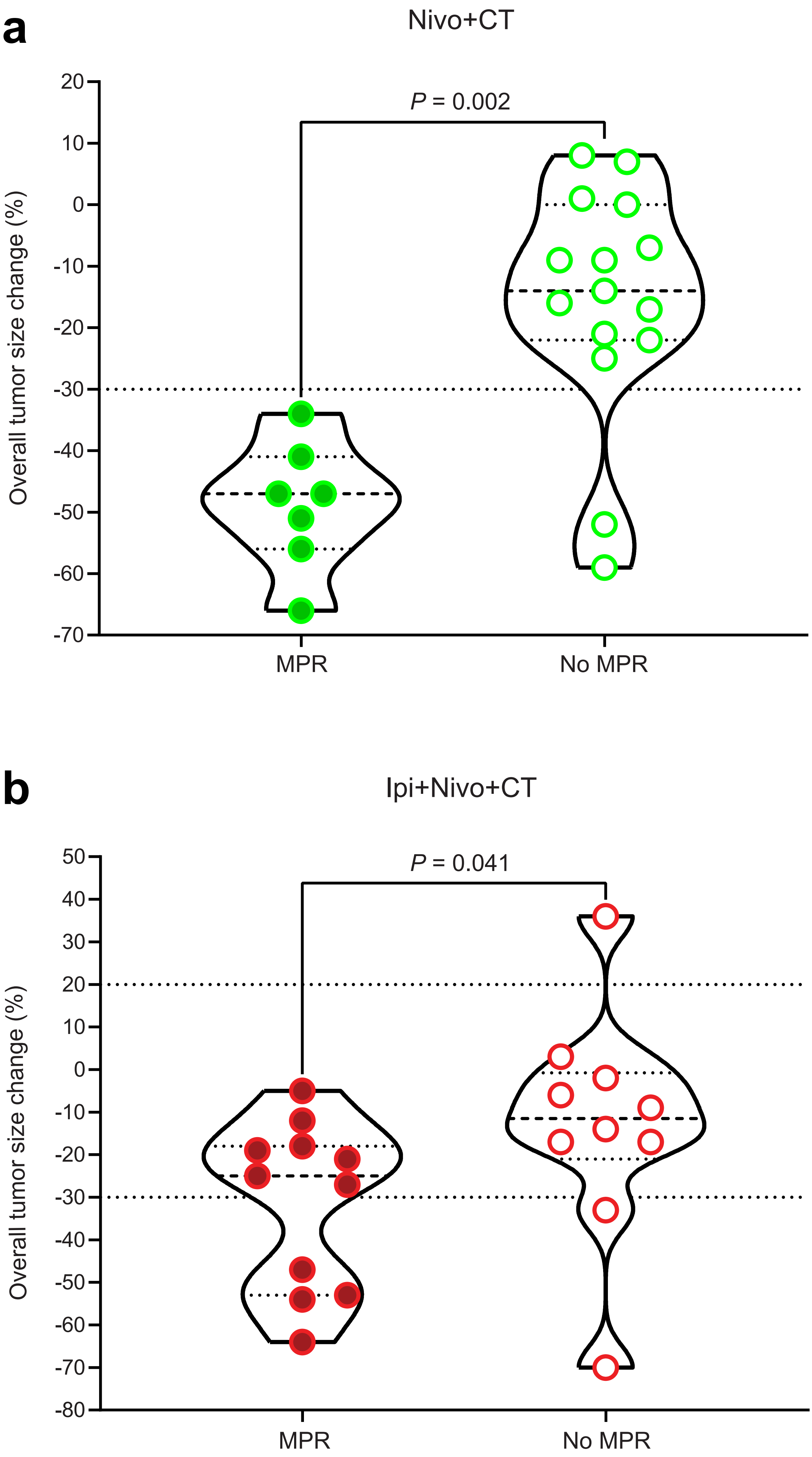

Supplementary Figure 1

Supplementary Figure 1. Overall tumor size change from baseline to post-therapy by MPR. **a**,**b**, Violin plots depict the association between overall tumor size change from baseline (pre-therapy) to post-therapy by MPR status in Nivo+CT (*n* = 22) (**a**) and Ipi+Nivo+CT (*n* = 21) (**b**). One patient in the Ipi+Nivo+CT arm was not evaluable due to death from SARS-CoV-2-related complications (non-treatment related). In **a**, the median percent change in tumor size from baseline to post-therapy for 22 patients was -21.5% (range -66% to 8%). In seven patients with MPR the median percent change was -47% (range -66% to -34%) as compared to -14% (range -59 to 8%) in 15 patients without MPR. In **b**, the median percent change in tumor size from baseline to post-therapy for 21 patients was -18% (range -70% to 36%). In 11 patients with MPR the median percent change was -25% (range -64% to -5%) as compared to -11.5% (range -70% to 36%) in ten patients without MPR. The green filled and empty circles depict data from MPR and No MPR, respectively, in Nivo+CT patients, and the red filled and empty circles depict data from MPR and No MPR, respectively, in Ipi+Nivo+CT patients. Data are presented as median with minima, lower and upper quartiles, and maxima using violin plots. The dashed line indicates the median; the dotted lines indicate the lower quartile and upper quartile values; top and bottom indicate the maxima and minima. Dashed line at 20% point depicts cutoff for PD. Dashed line at −30% point depicts cutoff for PR. Two-sided *P* value is from Wilcoxon rank-sum test. Nivo, Nivolumab; Ipi, Ipilimumab; CT, chemotherapy; MPR, major pathologic response.

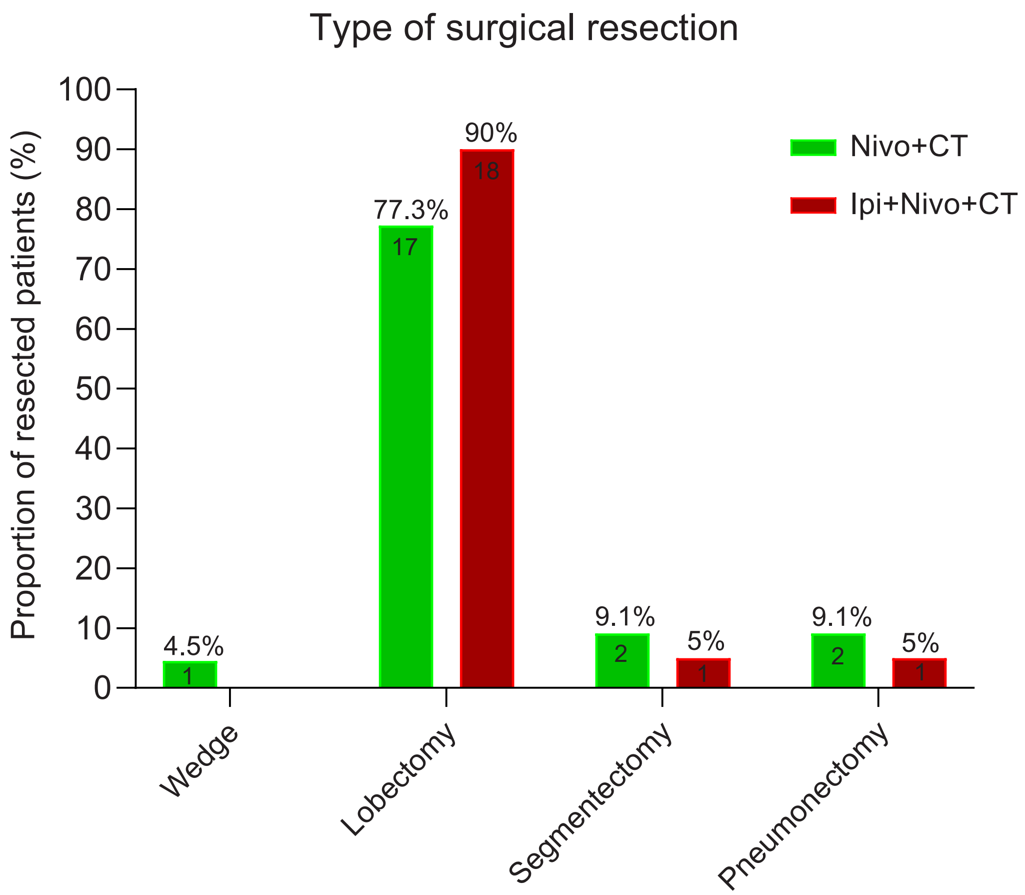

Supplementary Figure 2

Supplementary Figure 2. Type of surgical resection in both treatment arms. Type of surgical resection in Nivo+CT (green; *n* = 22) and Ipi+Nivo+CT (red; *n* = 20). Two patients in the Ipi+Nivo+CT arm were not resected due to death from SARS-CoV-2 infection-related complications (non-treatment related) in one patient and complexity of surgical resection and its associated risks in the second patient. Nivo, nivolumab; Ipi, ipilimumab; CT, chemotherapy.

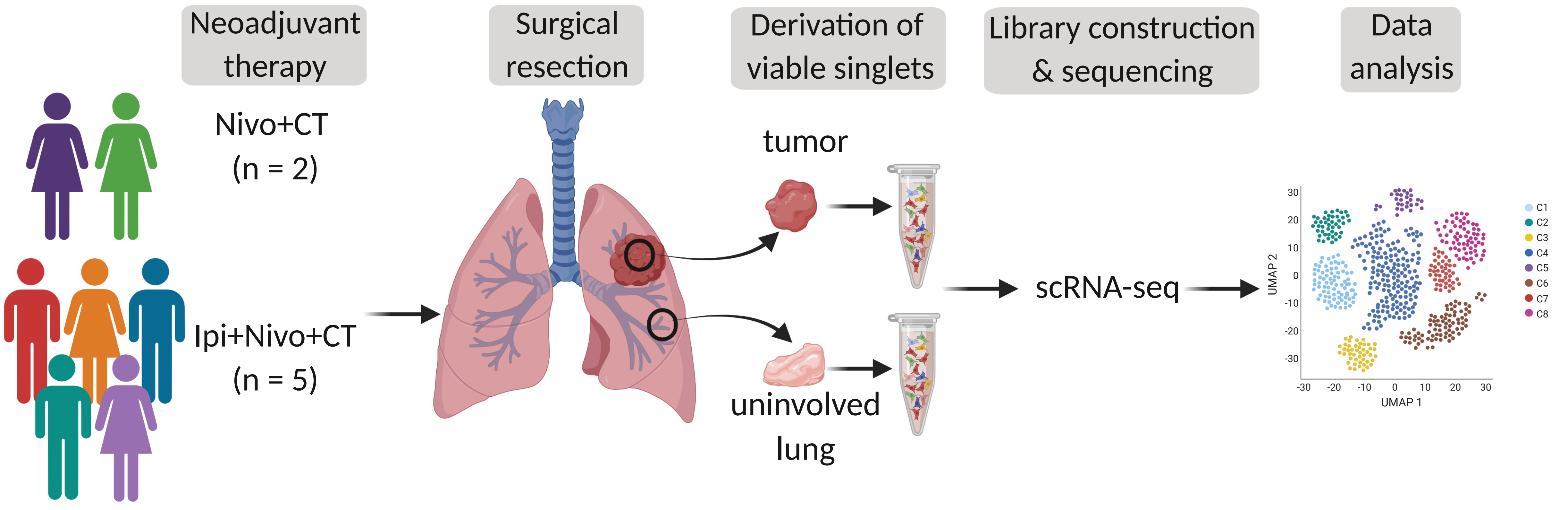

Supplementary Figure 3

Supplementary Figure 3. Single-cell RNA-sequencing analysis of surgically resected tissues from patients treated with neoadjuvant Nivo+CT and Ipi+Nivo+CT. Viable singlets were obtained and derived from surgically resected tumor and uninvolved normal tissues from two patients treated with neoadjuvant Nivo+CT and from five patients treated with neoadjuvant Ipi+Nivo+CT. One lymph node (LN) sample was also studied from a patient treated with Nivo+CT. Viable singlets from all samples (*n* = 15 samples) were then analyzed by single-cell RNA-sequencing (scRNA-seq) using the 10X Genomics platform to identify immune cell populations that may be implicated in different clinical and immune responses between both treatment arms. Nivo, nivolumab; Ipi, ipilimumab; CT, chemotherapy. BioRender (https://biorender.com) was used to generate this Figure.

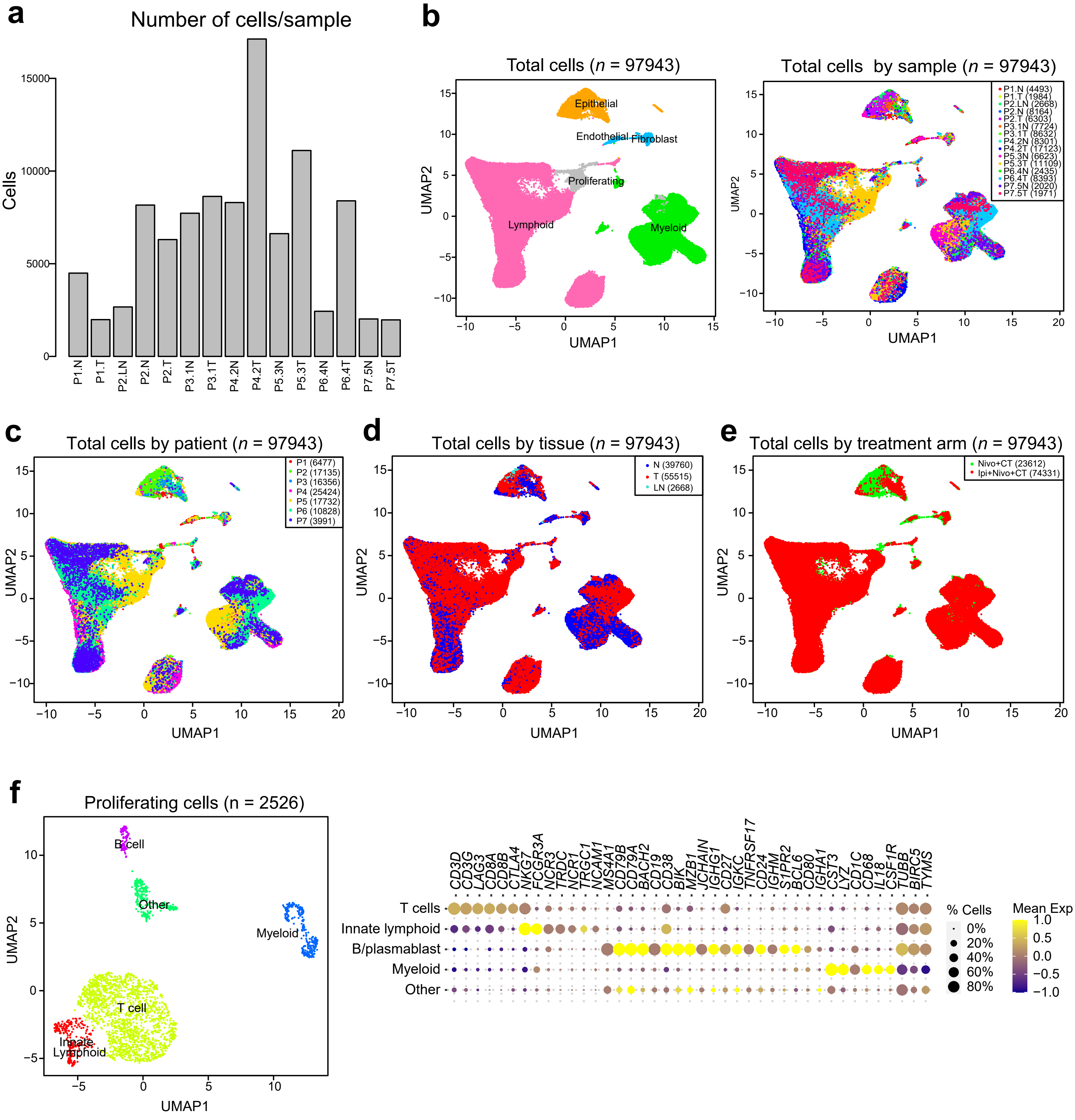

Supplementary Figure 4

Supplementary Figure 4. Visualization and clustering of 97,943 cells analyzed by single-cell RNA-sequencing. a, Barplot depicting numbers of sequenced cells in each of seven paired tumor and normal tissues and in a matched lymph node (LN) specimen from patient 2 (P2). Uniform manifold approximation and projection (UMAP) visualization of 97,943 high-quality cells that were retained for analysis and following clustering color-coded by cell lineage (**b, left**; lymphoid, myeloid, epithelial, stromal, and cycling/proliferating cells), sample (**b, right**), patient (**c**), tissue (**d**; tumor, normal, LN) and treatment arm (**e**; Nivo+CT, Ipi+Nivo+CT). **f**, Visualization of 2,526 cycling cells following clustering and color-coded by their lineages (left panel). Bubble plot is showing mean expression and abundance of marker genes that are differentially expressed among proliferating cells based on lineage (right panel). Nivo, nivolumab; Ipi, ipilimumab; CT, chemotherapy.

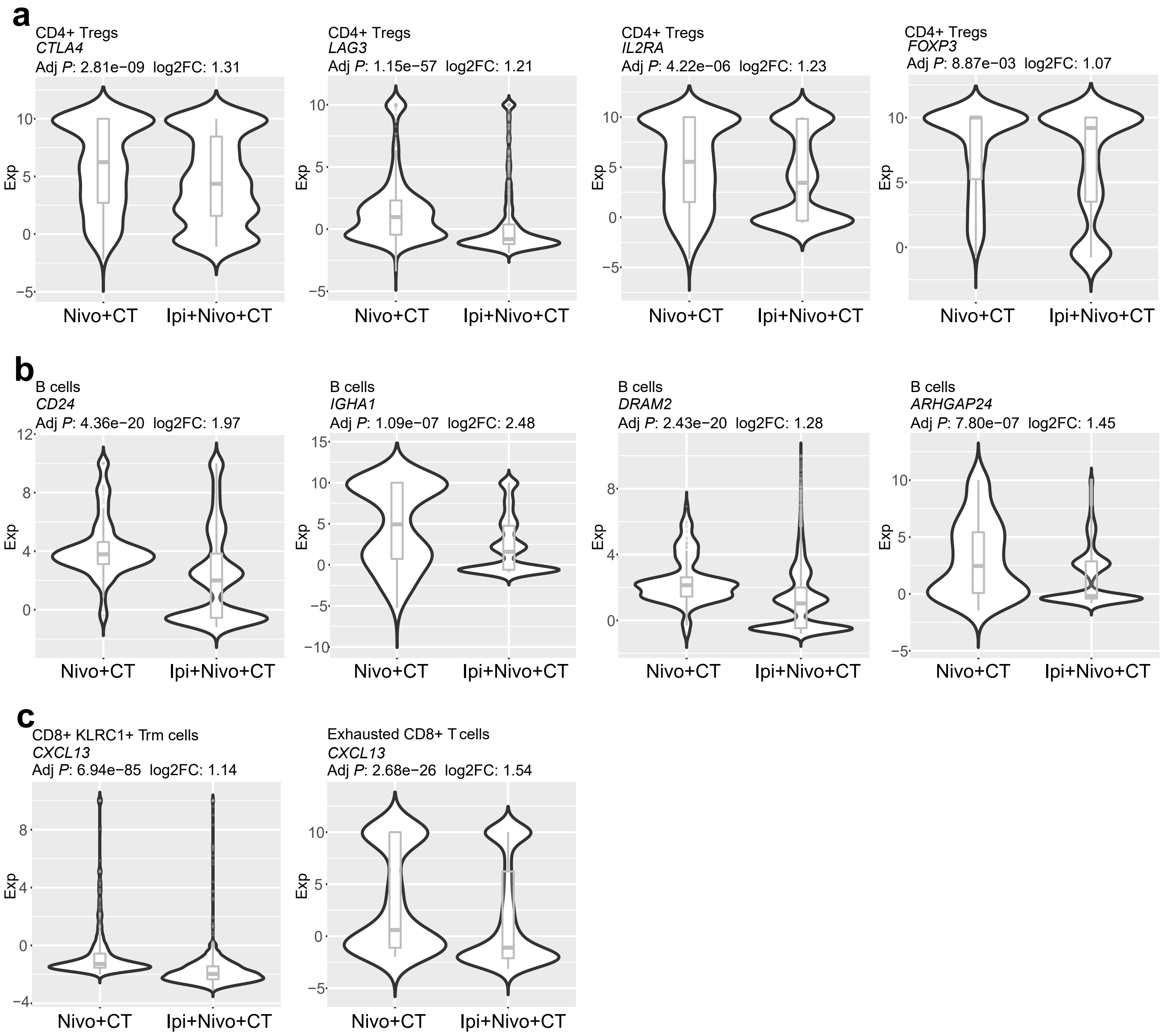

Supplementary Figure 5

Supplementary Figure 5. Differentially expressed immune cell-specific genes between NSCLCs from Nivo+CT- and Ipi+Nivo+CT-treated patients. Violin plots depicting genes that are differentially expressed in CD4+ T regulatory (Treg) (**a**), B (**b**), CD8+ KLRC1+ tissue resident memory (Trm) and exhausted CD8+ T (**c**) cells from Nivo+CT- and Ipi+Nivo+CT-treated tumors. *P* values were computed from two-sided Wilcoxon rank-sum tests and adjusted by false discovery rate (FDR). Boxes within violin plots represent the upper and lower quantile (75% and 25% percentile) of the data, the middle bar in the box represents the median (50% percentile), the tips of upper and lower whiskers represent the 95% and 5% percentile, respectively. Nivo, nivolumab; Ipi, ipilimumab; CT, chemotherapy.

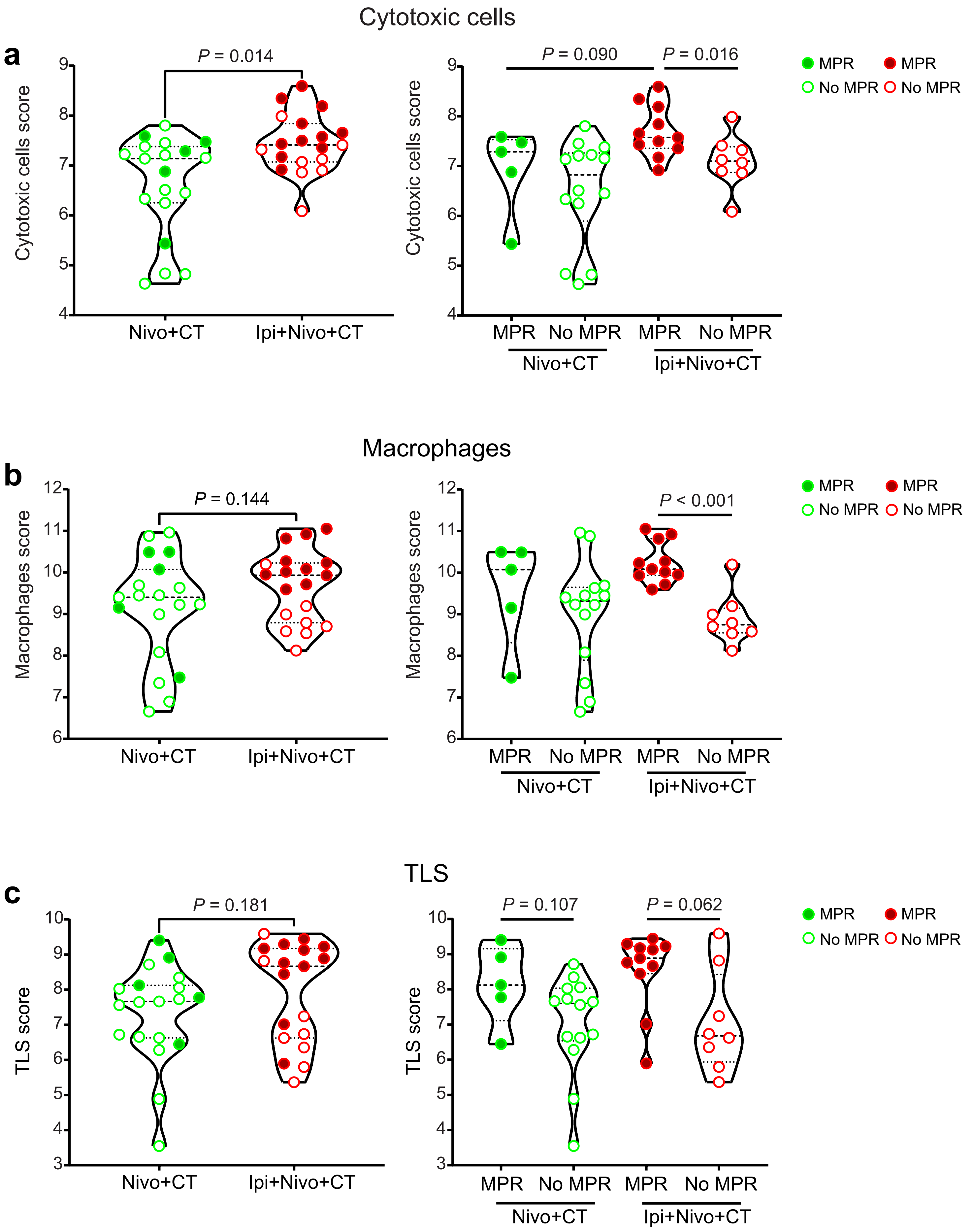

Supplementary Figure 6

Supplementary Figure 6. Composition of tumor immune infiltrates from patients treated with neoadjuvant Nivo+CT and Ipi+Nivo+CT. NanoString gene expression analysis was performed on post-treatment tumor tissue samples from patients in the Nivo+CT (n = 19) and Ipi+Nivo+CT (n = 19) arms. **a**–**c**, Violin plots show the distribution of cytotoxic cells (**a**), macrophages (**b**), and TLS (**c**) scores (log2 normalized counts) in resected tumors by treatment arm (left panels) and by treatment arm and response (MPR, No MPR) (right panels).The TLS signature score is derived from the median expression of *CD79A, MS4A1, LAMP3,* and *POU2AF1* genes. The green filled and empty circles depict data from MPR and No MPR, respectively, in Nivo+CT patients, and the red filled and empty circles depict data from MPR and No MPR, respectively, in Ipi+Nivo+CT patients. Data are presented as the median with minima, lower and upper quartiles, and maxima using violin plots. The dashed line indicates the median; the dotted lines indicate the lower quartile and upper quartile values; the top and bottom indicate the maxima and minima. Two-sided P values are from Wilcoxon rank-sum test in the left panels in **a** and **c** and in the right panels in **a**-**c**. Two-sided P value is from unpaired t-test in the left panel in **b**. Nivo, nivolumab; Ipi, ipilimumab; CT, chemotherapy. MPR, major pathologic response. Source data is provided as Source Data file.

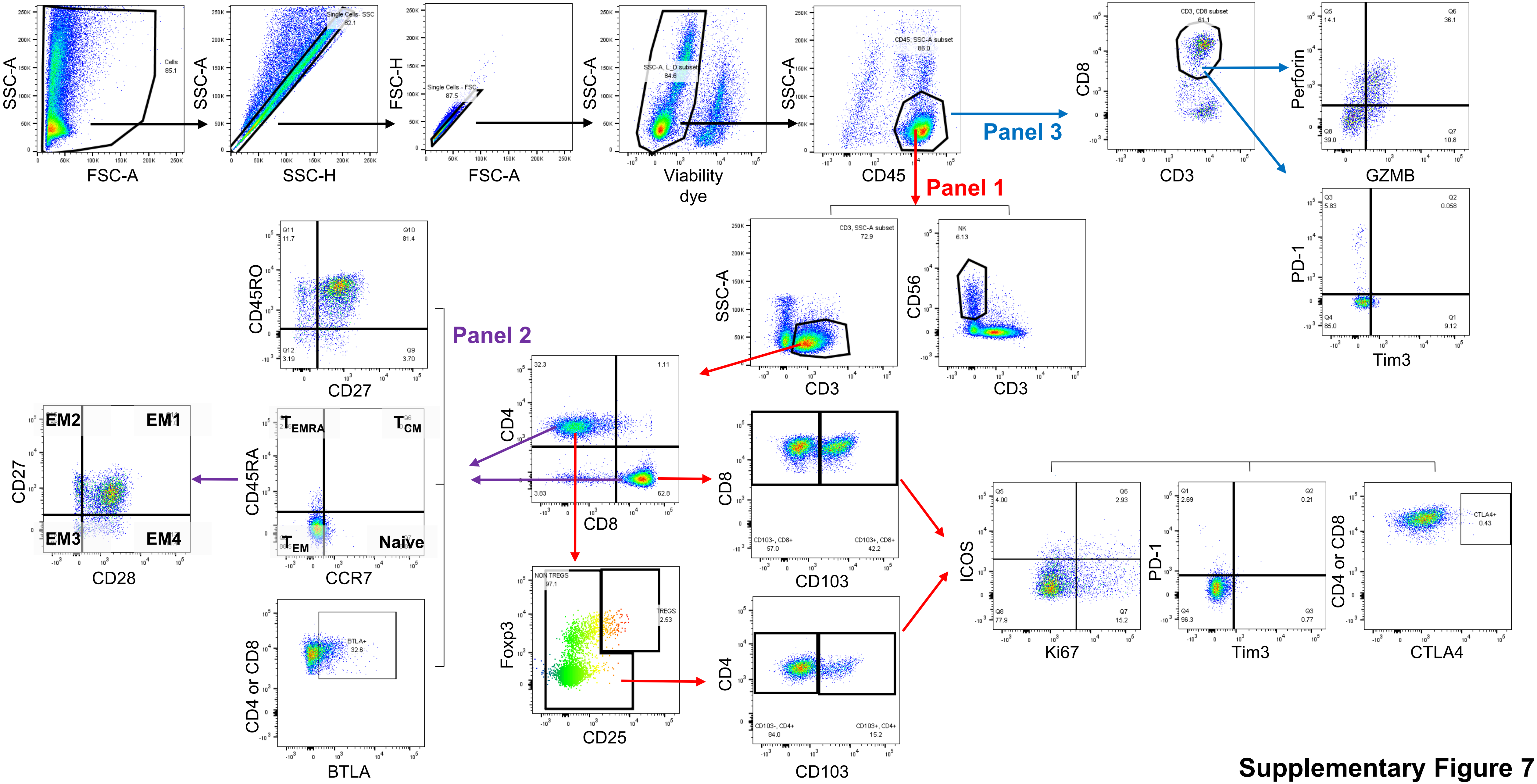

Supplementary Figure 7

Supplementary Figure 7. Flow cytometry gating strategy for T cell activation, T cell memory and T cell functional panels. The gating strategy is shown including initial QC gates (SSC singlets, FSC singlets, live cells) followed by immune cell subsets included in the panel. The frequencies referenced for each subgated cell population shown are from the parental gate for panel 1 (T cell activation), panel 2 (T cell memory), and panel 3 (T cell functional molecules). Experiments and gating related to presented results were conducted once. Subgating was only performed when more than 100 events were present in parental gate.
