# Supplementary material for: Neoadjuvant chemotherapy plus nivolumab with or without ipilimumab in operable non-small cell lung cancer: the phase 2 platform NEOSTAR trial
Source: Nat Med. 2023 Mar 16;29(3):593–604. doi: 10.1038/s41591-022-02189-0 (PMC10033402; doi:10.1038/s41591-022-02189-0)
Supplement: Supplementary file 2 — Reporting Summary [file 41591_2022_2189_MOESM2_ESM.pdf]

nature portfolio

Corresponding author(s): Tina Cascone

Last updated by author(s): Dec 14, 2022

Reporting Summary

Nature Portfolio wishes to improve the reproducibility of the work that we publish. This form provides structure for consistency and transparency in reporting. For further information on Nature Portfolio policies, see our Editorial Policies and the Editorial Policy Checklist.

Statistics

For all statistical analyses, confirm that the following items are present in the figure legend, table legend, main text, or Methods section.

n/a	Confirmed	
☐	☒	The exact sample size (*n*) for each experimental group/condition, given as a discrete number and unit of measurement
☐	☒	A statement on whether measurements were taken from distinct samples or whether the same sample was measured repeatedly
☐	☒	The statistical test(s) used AND whether they are one- or two-sided<br>*Only common tests should be described solely by name; describe more complex techniques in the Methods section.*
☒	☐	A description of all covariates tested
☐	☒	A description of any assumptions or corrections, such as tests of normality and adjustment for multiple comparisons
☐	☒	A full description of the statistical parameters including central tendency (e.g. means) or other basic estimates (e.g. regression coefficient) AND variation (e.g. standard deviation) or associated estimates of uncertainty (e.g. confidence intervals)
☐	☒	For null hypothesis testing, the test statistic (e.g. *F*, *t*, *r*) with confidence intervals, effect sizes, degrees of freedom and *P* value noted<br>*Give P values as exact values whenever suitable.*
☐	☒	For Bayesian analysis, information on the choice of priors and Markov chain Monte Carlo settings
☒	☐	For hierarchical and complex designs, identification of the appropriate level for tests and full reporting of outcomes
☐	☒	Estimates of effect sizes (e.g. Cohen's *d*, Pearson's *r*), indicating how they were calculated

Our web collection on statistics for biologists contains articles on many of the points above.

Software and code

Policy information about availability of computer code

Data collection

Clinical Data were collected via the Data Management Initiative (DMI) project at the University of Texas MD Anderson Cancer Center and Microsoft Excel (v. 2016) Spreadsheets.

Pathological data collection was performed with routine cytophatological/histopathological processing of tissue samples as detailed in the Methods session "Pathological analysis" of the manuscript. Data were collected using Microsoft Excel v.2016

Single-cell RNA-sequencing (scRNA-seq): Available tumor and matched uninvolved lung tissues were freshly collected at surgery and placed in ice-cold DMEM medium supplemented with 2% FBS and immediately minced and enzymatically digested in DMEM containing 0.16 mg/mL DNase I (9003-98-9; Worthington Biochemical Corp.) and 328 U/mL Liberase (5401020001; Roche) for 30 minutes at 37°C. Lysate was filtered and washed, after which red blood cells were eliminated using Red Blood lysis buffer (A1049201; Gibco). Total cells were cryopreserved in FBS with 10% DMSO and stored in the vapor phase of a liquid nitrogen tank until further processing. Details of scRNA-seq library preparation are available in the manuscript.

NanoString data: Available tumor samples were collected post neoadjuvant treatments at surgery. FFPE tissue samples from resected tumors were cut into 4 µm thick sections. Tissue sections were processed and with reagents and methods described in the manuscipt. RNA quality and quantity were assessed using the Nanodrop spectrometer (ND-Nanodrop1000, Thermo Scientific, Wilmington, MA, USA). Data were collected using Microsoft Excel v.2016.

Immunohistochemistry (IHC) of PD-L1 data: Available FFPE tumor tissue samples were collected from patients pre-neoadjuvant therapies for single chromogenic IHC staining of tumor PD-L1 using a a Leica Bond Max autostainer system (Leica Biosystems). Automated standard Leica protocol and reagents were utilized as detailed in the methods of the manuscript. Data were collected using Microsoft Excel v.2016.

Multiparameter flow cytometry data: Fresh uninvolved tumor-adjacent and tumor tissues collected at surgery were disaggregated using the BD Medimachine System (BD Biosciences) to make a single cell suspension for flow cytometry staining. Detailed methods and antibodies are

described in the manuscript and in the antibody section below. Data were acquired with the Fortessa X20 (BD Bioscience) or Canto II (BD Bioscience) using BD FACSDiva software v8.0.1. and Microsoft Excel (v. 2016).

Multiplex immunofluorescence (mIF) staining data: Using an automated staining system (BOND-RX; Leica Microsystems), 4-µm-thick FFPE tumor sections were stained for two panels containing antibodies against the antibodies in panel 1 and panel 2, as detailed in the manuscript methods and in the antibody section below. The stained slides were scanned using the multispectral microscope, Vectra v.3.0.3 imaging system (Akoya Biosciences/ PerkinElmer). After the slides were scanned in low magnification, a pathologist selected around five regions of interest (ROIs; each ROI: 0.3345mm2) per sample to cover around 1.65 mm2 of tumor tissue using the phenochart v.1.0.9 viewer (Akoya Biosciences/PerkinElmer). The data were collected using Microsoft Excel v.2016.

Gut microbiome data: Fecal samples were collected from patients on NEOSTAR trial as previously reported (https://pubmed.ncbi.nlm.nih.gov/33603241/). Total DNA was extracted from fecal samples using the QIAamp DNA Stool Kit (Qiagen, Hilden, Germany), followed by a bead-beating lysis step.

Data analysis

Statistics: As the primary analysis, an uniformly minimum variance unbiased estimator (UMVUE) of the MPR rate was obtained using the approach proposed by Jung and Kim within each study arm. A P value for the statistical test against the assumed historical control of 15% and the corresponding 80% two-sided confidence interval (CI) was calculated using the method developed by Koyama & Chen to adjust for the Simon’s two-stage design’s adaptiveness. The distributions of EFS and OS were estimated by the Kaplan-Meier method. Logrank test was performed to test the difference in survival between groups. A two-sided P value of 0.05 was considered significant. Analyses were performed in SAS v.9.4, R v.4.1.2 and GraphPad Prism v.9.0.0.

scRNA-seq: Sequencing was performed using the NovaSeq 6000 platform from Illumina. The raw reads were aligned to human reference genome GRCh38 (hg38) and processed by 10X Genomics Cell Ranger V3.1.0 to generate the unique molecular identifier (UMI) count data matrix. The UMI data matrix were processed using the Seurat package (V3) , with the following workflow: 1) Data filtering: the UMI data matrix were filtered to remove genes that have zero total UMI counts in all cells, as well as cells with less than 300 expressed genes or more than 10% of total UMI count of mitochondrial genes, 2) Data normalization and integration: Filtered UMI data matrices from different data batches were normalized, scaled, batched corrected, and integrated using the data integration workflow in Seurat v3 with the integration anchor features set to all genes in filtered datasets as described by Stuart and colleagues (in Stuart et al, Cell, 2019). 3) Data reduction and visualization: principal component analysis (PCA) were performed using highly variable genes identified by the Seurat “VariableFeatures” function. The top ranked principal components that covered 80% of the total variance were selected and transformed into UMAP components for visualization. 4) Unsupervised clustering: cell clusters were identified using Seurat's “FindClusters” function, with resolution value manually adjusted to find the best separation. 5) Cluster annotation: The marker genes for each cluster were identified using Seurat's “FindClusterMarkers” function. These markers genes, combined with markers for known cell types such as immune cells and epithelial cells etc., were used to identify the major cell lineages of each cluster. Each cell lineage was further clustered to identify sub-lineages a if needed. During these processes additional doublets were identified and removed from the clusters. These clustering/identification processes were performed iteratively until all cell populations were annotated. 6) Differential analysis: for each cell population, we identified the differential expressed genes between sample types (tumor vs uninvolved) and treatment group (Nivo+CT vs Ipi+Nivo+CT), using Wilcoxon rank-sum test, with statistical cutoff set to false discovery rate (FDR) less than 0.05 and log2 fold change greater than 1. The proportions of sub lineages between sample types (tumor vs uninvolved) and treatment group (Nivo+CT vs Ipi+Nivo+CT) were compared using two-sided proportion test. All statistical analyses were performed in R v4.0.1. Codes used for scRNA-seq analysis are available from https://github.com/MD-Anderson-Bioinformatics/Neoimmuno.

Immunohistochemistry analysis: PD-L1 stained slides were scored by standard microscopy following the recommendations of the International Association for the Study of Lung Cancer guidelines (PMID: 29800747). The results were plotted using GraphPad Prism v.9.00.

Flow Cytometry analysis: Data were analyzed using FlowJo Software v.10.5.3 (Tree Star, Inc.). Dead cells were stained using LIVE/DEAD Fixable Yellow Dead Cell Stain dye (catalog no. L-34968, Life Technologies) and excluded from the analysis. Analyzed data were plotted using GraphPad prism v. 9.00.

Multiplex immunofluorescence analysis: ROIs were analyzed by a pathologist using InForm v.2.8.2 image analysis software (Akoya Biosciences). All the data were consolidated using the R studio v.3.5.3 (Phenopter v.0.2.2 packet, Akoya Biosciences/PerkinElmer) and SAS v.7.1 Enterprise. The data were plotted using GraphPad Prism v.9.00.

NanoString analysis: nCounter Digital Analyzer was used to tabulate the counts of the reporter probes and for further analysis raw data output was imported into nSolver analysis software (v4.0.70) (http://www.nanostring.com/products/nSolver). Normalization, cell type and differential gene expression analyses were performed using the nSolver Advanced data analysis package (v2.0.134). The data were plotted using GraphPad prism v. 9.0.0.

Gut microbiome analysis: The V4 region of the bacterial 16S rRNA gene was amplified and sequenced on the Illumina MiSeq (Illumina, Inc.) platform using the 2x250 bp paired-end protocol yielding paired-end reads with near-complete overlap. Raw FASTQ files were processed using DADA2 (1.18) to generate amplicon sequence variants (ASVs) and taxonomies assigned with SILVA database v138 (https://www.arb-silva.de). The resulting ASV table and taxonomies were used to compute alpha and beta diversity metrics as well as taxonomic relative abundances. The sequencing depths ranged from 19,310 to 159,961 with a mean of 59,057 reads per sample. Alpha diversity was calculated using Shannon Index. Bray-Curtis dissimilarity were used to calculate the pairwise dissimilarities and perform principal coordinate analysis (PCoA) between samples. PERMANOVA analyses (with 999 permutations) and beta-dispersion tests were used to compare microbiota diversity and dispersion between the two trials. Differentially abundant taxa were identified in each of the trials using the statistical method implemented in the R package DESeq2. The results were plotted in R (R Core Team 2020; https://www.R-project.org) using ggplot2 package (https://ggplot2.tidyverse.org).

For manuscripts utilizing custom algorithms or software that are central to the research but not yet described in published literature, software must be made available to editors and reviewers. We strongly encourage code deposition in a community repository (e.g. GitHub). See the Nature Portfolio guidelines for submitting code & software for further information.

Data

Policy information about availability of data

All manuscripts must include a data availability statement. This statement should provide the following information, where applicable:

- Accession codes, unique identifiers, or web links for publicly available datasets
- A description of any restrictions on data availability
- For clinical datasets or third party data, please ensure that the statement adheres to our policy

De-identified single-cell RNA-sequencing raw data reported in this manuscript have been deposited in the European Genome-phenome Archive with accession number EGAS00001006728. Access to this dataset is controlled by the institutional Data Access Committee in compliance with the NIH policy for Data Management and Sharing and in accordance with an alliance agreement between MD Anderson Cancer Center and Bristol Myers Squibb. Access to this dataset will be granted upon review and acceptance of academic requests. Further information about EGA can be found at https://egaarchive.org. The raw reads were aligned to human reference genome GRCh38 (hg38). The 16S fecal microbiome sequencing data have been deposited in the National Center for Biotechnology Information Sequence Read Archive under the SRA BioProject ID PRJNA665109 (https://www.ncbi.nlm.nih.gov/bioproject/PRJNA665109). Taxonomies were assigned with SILVA database v138 (https://www.arb-silva.de). Source data for Figure 5, Extended Data Figures 7-10 and Supplementary Figure 6 are provided.

Field-specific reporting

Please select the one below that is the best fit for your research. If you are not sure, read the appropriate sections before making your selection.

☒ Life sciences ☐ Behavioural & social sciences ☐ Ecological, evolutionary & environmental sciences

For a reference copy of the document with all sections, see nature.com/documents/nr-reporting-summary-flat.pdf

Life sciences study design

All studies must disclose on these points even when the disclosure is negative.

Sample size	Simon's minimax two-stage design was applied to test the major pathologic response rate for each one of the two treatment arms. We assumed the 15% major pathologic response rate under the null hypothesis versus the 40% major pathologic response rate under the alternative hypothesis. For each treatment arm, 15 patients were enrolled in the first stage. If only two or less of the 15 patients have major pathologic response, enrollment to that treatment arm would be terminated and the treatment is considered inefficacious. Otherwise, with at least three major pathologic response, additional 6 patients would be enrolled to reach a total of 21 patients. At the end of trial, if we observe 6 or more patients have major pathologic response, the treatment is considered efficacious and inefficacious otherwise. The trial would have 90% power when the major pathologic response rate is 40%. When the major pathologic response rate is 15%, the probability of early termination is 0.60 with an average sample size of 17.4 and one-sided 10% type I error rate.
Data exclusions	Clinical analyses: All eligible patients enrolled into the study were included in the analyses.<br><br>Correlative analyses: All samples available and considered appropriate based on QC for correlative studies at time of analyses were included.<br><br>Flow cytometry analysis: Available samples were excluded from analysis if they did not pass the respective QC for a given assay as detailed in Methods and Figure Legends of the manuscript.
Replication	Replication was not applicable to this study as this was a clinical study with unique patient samples. All techniques and reagents used for the correlative analyses of this study had been previously optimized and validated.
Randomization	Patient were enrolled to the nivolumab plus chemotherapy arm followed by ipilimumab plus nivolumab plus chemotherapy arm of the NEOSTAR platform study. Randomization was not performed in these two arms. Treatment allocation was not relevant in this multi-arm platform trial with two independent single-arm. These two arms were expected to be analyzed and reported separately with the goal to expedite the investigation of novel immunotherapy-based strategies in the neoadjuvant setting.
Blinding	The trial was not a blinded study. Blinding was not practical in this multi-arm platform phase 2 trial of single studies performed in sequence as experimental treatments were administered intravenously with different doses and schedules. However, after initial clinical reporting, the primary endpoint of the study was reviewed in a blinded manner by two pathologists experienced in the evaluation of tumor response after neoadjuvant therapy. Furthermore, the study was designed to compare the primary endpoint to historical controls of neoadjuvant chemotherapy and both experimental arms were novel strategies added to standard-of-care approach.

Reporting for specific materials, systems and methods

We require information from authors about some types of materials, experimental systems and methods used in many studies. Here, indicate whether each material, system or method listed is relevant to your study. If you are not sure if a list item applies to your research, read the appropriate section before selecting a response.

Materials & experimental systems

n/a	Involved in the study
	☒ Antibodies
☒	Eukaryotic cell lines
☒	Palaeontology and archaeology
☒	Animals and other organisms
	☒ Human research participants
	☒ Clinical data
☒	Dual use research of concern

Methods

n/a	Involved in the study
☒	ChIP-seq
	☒ Flow cytometry
☒	MRI-based neuroimaging

Antibodies

Antibodies used

Immunohistochemistry (IHC) studies for PD-L1 staining in malignant cells: PD-L1 anti-human antibody clone 28-8, catalog no. ab205921, dilution 1:100; Abcam, Cambridge, MA, USA.

Flow Cytometry studies: fluorochrome-conjugated monoclonal antibodies against CD45 (BUV395, Clone HI30, catalog no. 563792, BD Biosciences, 5µl/sample), CD3 (PerCP-Cy5.5, clone SK7, catalog no. 340949, BD Biosciences, 10µl/sample), CD8 (AF700, clone RPA-T8, catalog no. 557945, BD Biosciences, 5µl/sample), CD4 (BUV496, clone SK3, catalog no. 612936, BD Biosciences, 5µl/sample), PD-1 (SB645, clone MIH4, catalog no. 64-9969-42, eBioscience, 4µl/sample), TIM3 (BV605, Clone F38-2E2, catalog no. 345018, BioLegend, 4µl/sample), CD103 (BV711, clone Ber-Act8, catalog no. 563162, BD Biosciences, 5µl/sample), CTLA-4 (BV786, clone BNI3, catalog no. 563931, BD Biosciences, 3µl/sample), GITR (AF488, clone eBioAITR, catalog no. 53-5875-42, eBioscience, 5µl/sample), LAG3 (PE, clone 3DS223H, catalog no. 12-2239-42, eBioscience, 5µl/sample), CD56 (PE-Cy7, clone B159, catalog no. 557747, BD Biosciences, 5µl/sample), ICOS (BV421, clone C398.A4, catalog no. 313524, BioLegend, 5µl/sample) and CD25 (APCFire/750, clone BC96, catalog no. 302642, BioLegend, 5µl/sample), FOXP3 (PE-eFluor610, clone PCH101, catalog no. 61-4776-42, eBioscience, 5µl/sample) and Ki67 (APC, clone 20Raj1, catalog no. 17-5699-42, eBioscience, 5µl/sample), CD27 (FITC, clone M-T271, catalog no. 555440, BD Biosciences, 20µl/sample), CCR7 (PerCP-Cy5.5, clone 150503, catalog no. 561144, BD Biosciences, 5µl/sample), CD45RA (V450, clone HI100, catalog no. 560362, BD Bioscience, 5µl/sample), CD3 (APC, clone UCHT1, catalog no. 555335, BD Biosciences, 20µl/sample), CD4 (BUV496, clone SK3, catalog no. 612936, BD Biosciences, 5µl/sample), CD8 (AF700, clone RPA-T8, catalog no. 557945, BD Biosciences, 5µl/sample), CD45RO (APC-H7, clone UCHL1, catalog 561137, BD Biosciences, 5µl/sample), BTLA (PE, clone J168-540, catalog no. 558485, BD Biosciences, 5µl/sample), and CD28 (PE-Cy7, Clone CD28.2, catalog no. 560684, BD Biosciences, 5µl/sample), PD-1 (PerCP-Cy5.5, clone EH12, catalog no. 329914, BioLegend, 5µl/sample), TIM3 (APC, clone F38-2E2 catalog no. 17-3109-42, eBioscience, 5µl/sample), CD8 (APC-Cy7, clone RPA-T8, catalog no. 557760, BD Biosciences,3µl/sample), and CD3 (PE-Cy7, clone UCHT1, catalog no. 563423 BD Biosciences, 5µl/sample), Perforin (FITC, clone DG9, catalog no. 11-9994-42, eBiosciences, 5µl/sample), Granzyme B (V450, clone GB11, catalog no. 561151, BD Biosciences, 5µl/sample), and IFNγ (PE, clone B27, catalog no. 559327, BD Biosciences, 10µl/sample) anti-human antibodies. Dead cells were stained using LIVE/DEAD Fixable Yellow Dead Cell Stain dye (catalog no. L-34968, Life Technologies, 1µl/sample).

Multiplex Immunofluorescence studies: antibodies against (Panel 1): cytokeratin (clone AE1/AE3, catalog no. M351501-2, dilution 1:300, Dako, Santa Clara, CA), CD3 (catalog no. IS503, dilution 1:100, Dako), CD8 (clone C8/144B, catalog no. MS-457-S, dilution 1:300, Thermo Fisher Scientific), CD68 (clone PG-M1, catalog no. M0875, dilution 1:450, Dako), PD-1 (clone EPR4877-2, catalog no. ab137132, dilution 1:250, Abcam), and PD-L1 (clone E1L3N, catalog no. 13684S, dilution 1:3,000, Cell Signaling Technology); and antibodies against (Panel 2): panel 2: cytokeratin (clone AE1/AE3, catalog no. M351501-2, dilution 1:300, Dako), CD3 (catalog no. IS503, dilution 1:100, Dako), CD8 (clone C8/144B, catalog no. MS-457-S, dilution 1:300, Thermo Fisher Scientific), CD45RO (clone UCHL1, catalog no. PA0146, Cell Signaling Technology), Granzyme B (clone 11F1, catalog no. PA0291, Cell Signaling Technology) and FOXP3 (clone D2W8E, catalog no. 98377S, Cell Signaling Technology). All the markers were stained in sequence using their respective fluorophore containing in the Opal 7 kit (catalog no. NEL797001KT; Akoya Biosciences/PerkinElmer).

Validation

Immunohistochemistry (IHC) antibody for PD-L1 staining in malignant cells was previously validated as reported in https://www.ncbi.nlm.nih.gov/pubmed/28719380.

Flow cytometry staining:: antibodies were titrated on PBMCs and expanded tumor-infiltrating lymphocytes including unstained controls. The majority of these markers have been previously described in Bentebibel et al., Cancer Discovery 2019 (PMID:30988166).
CD45 (BUV395, Clone HI30, Cat. No. 563792) - antibody internally validated by using tumor cells as a negative control and normal donor PBMCs as a positive control. Vendor validation and technical information can be found at https://www.bdbiosciences.com/content/bdb/paths/generate-tds-document.us.563792.pdf.
CD3 (PerCP-Cy5.5, Clone SK7, Cat. No. 340949, BD Biosciences [panel 1]; APC, Clone UCHT1, Cat. No. 555335, BD Biosciences [panel 2]; PE-Cy7, Clone UCHT1, Cat. No. 563423, BD Biosciences [panel 3]) - antibody internally titrated and validated using normal donor PBMCs and B cells as a negative control. Vendor validation and technical information can be found at https://www.bdbiosciences.com/content/bdb/paths/generate-tds-document.us.340949.pdf; https://www.bdbiosciences.com/content/bdb/paths/generate-tds-document.us.555335.pdf,;https://www.bdbiosciences.com/content/bdb/paths/generate-tds-document.us.563423.pdf.
CD8 (AF 700, Clone RPA-T8, Cat. No. 557945, BD Biosciences [panel 1 and panel 2]; APC-Cy7, Clone RPA-T8, Cat. No. 557760 [panel 3]) - antibody internally titrated and validated using expanded tumor infiltrating lymphocytes and normal donor PBMCs. B cells were used as a negative control from normal donor PBMCs; Vendor validation and technical information can be found at https://www.bdbiosciences.com/content/bdb/paths/generate-tds-document.us.557945.pdf; https://www.bdbiosciences.com/content/bdb/paths/generate-tds-document.us.557760.pdf.
CD4 (BUV496, Clone SK3, Cat. No. 612936, BD Biosciences [panel 1 and panel 2]) - antibody internally titrated and validated using expanded tumor-infiltrating lymphocytes and normal donor PBMCs. B cells were used as a negative control from normal donor PBMCs; Vendor validation and technical information can be found at https://www.bdbiosciences.com/content/bdb/paths/generate-tds-document.us.612936.pdf.

PD1 (SB645, Clone MIH4, Cat. No. 64-9969-42, eBiosciences [panel 1], PerCP-Cy5.5, Clone EH12 Cat. No. 329914, BioLgend [panel 3]) - antibody internally titrated and validated with respect to differential staining patterns on CD8 T cells from expanded tumor-infiltrating lymphocytes as a positive control and normal donor PBMCs as a negative control; Vendor validation and technical information can be found at https://www.thermofisher.com/antibody/product/CD279-PD-1-Antibody-clone-MIH4-Monoclonal/64-9969-42; https://www.biolegend.com/en-us/products/percp-cyanine5-5-anti-human-cd279-pd-1-antibody-5611
TIM3 (BV605, Clone F38-2E2, Cat. No. 345018, BioLegend [panel 1]; APC, Clone F38-2E2, Cat. No. 17-3109-42, eBioscience [panel 3]) - antibody internally titrated and validated with respect to differential staining patterns on CD8 T cells from expanded tumor-infiltrating lymphocytes as a positive control and normal donor PBMCs as a negative control; Vendor validation and technical information can be found at https://www.biolegend.com/en-us/products/brilliant-violet-605-anti-human-cd366-tim-3-antibody-8606; https://www.thermofisher.com/antibody/product/CD366-TIM3-Antibody-clone-F38-2E2-Monoclonal/17-3109-42.
CD103 (BV711, Clone Ber-Act8, Cat. No. 563162, BD Biosciences) - antibody internally validated by gating on T cells from normal donors as a negative control and CD8+ tumor-infiltrating lymphocytes as a positive control; Vendor validation and technical information can be found at https://www.bdbiosciences.com/content/bdb/paths/generate-tds-document.us.563162.pdf.
CTLA4 (BV786, Clone BNI3, Cat. No. 563931, BD Biosciences) - antibody internally validated with respect to differential staining pattern on activated T cells as compared to unactivated T cells from expanded tumor-infiltrating lymphocytes and normal donor PBMCs; Vendor validation and technical information can be found at https://www.bdbiosciences.com/content/bdb/paths/generate-tds-document.us.563931.pdf.
GITR (AF 488, Clone eBioAITR, Cat. No. 53-5875-42, eBioscience) - antibody internally validated by differential staining on unstimulated T cells from normal donor PBMCs as compared to expression on CD4+CD25+FoxP3+ tumor-infiltrating lymphocytes; Vendor validation and technical information can be found at https://www.thermofisher.com/antibody/product/CD357-AITR-GITR-Antibody-clone-eBioAITR-Monoclonal/53-5875-42.
LAG3 (PE, Clone 3DS223H, Cat. No. 12-2239-42, eBioscience) - antibody internally validated with respect to differential staining pattern on CD8 T cells and CD4 T cells from expanded tumor-infiltrating lymphocytes as a positive control as compared to from normal donor PBMCs as a negative control; Vendor validation and technical information can be found at https://www.thermofisher.com/antibody/product/CD223-LAG-3-Antibody-clone-3DS223H-Monoclonal/12-2239-42.
CD56 (PE-Cy7, Clone B159, Cat. No. 557747, BD Biosciences) - antibody internally validated by gating on CD3 negative cells from normal donor PBMCs; Vendor validation and technical information can be found at https://www.bdbiosciences.com/content/bdb/paths/generate-tds-document.us.557747.pdf.
ICOS (BV421, Clone C398.A4, Cat. No. 313524, BioLegend) - antibody internally validated with respect to differential staining pattern on activated T cells as compared to unactivated T cells from expanded tumor-infiltrating lymphocytes and normal donor PBMCs; Vendor validation and technical information can be found at https://www.biolegend.com/en-us/products/brilliant-violet-421-anti-human-mouse-rat-cd278-icos-antibody-8876.
CD25 (APCFire/750, Clone BC96, Cat. No. 302642, BioLegend) - antibody internally validated by assessing differential expression on activated and non-activated T cells; Vendor validation and technical information can be found at https://www.biolegend.com/en-us/products/apc-fire-750-anti-human-cd25-antibody-13841.
FOXP3 (PE-eFluor610, Clone PCH101, Cat. No. 61-4776-42, eBioscience) - antibody internally validated by gating on CD45+CD3+CD4+ T cells from normal donor PBMCs;Vendor validation and technical information can be found at https://www.thermofisher.com/antibody/product/FOXP3-Antibody-clone-PCH101-Monoclonal/61-4776-42.
Ki67 (APC, Clone 20Raj1, Cat. No. 17-5699-42, eBioscience) - antibody internally validated by gating on T cells from normal donor PBMCs as a negative control and expanded tumor-infiltrating lymphocytes as a positive control; Vendor validation and technical information can be found at https://www.thermofisher.com/antibody/product/Ki-67-Antibody-clone-20Raj1-Monoclonal/17-5699-42.
CD27 (FITC, Clone M-T271, Cat. No. 555440, BD Biosciences) - antibody internally validated by gating on T cells from normal donor PBMCs as a positive control and monocytes as a negative control; Vendor validation and technical information can be found at;https://www.bdbiosciences.com/content/bdb/paths/generate-tds-document.us.555440.pdf.
CCR7 (PerCP-Cy5.5, Clone 150503, Cat. No. 561144, BD Biosciences) - antibody internally validated by gating on T cells from normal donor PBMCs as a positive control and fluorescence minus one negative controls; Vendor validation and technical information can be found at https://www.bdbiosciences.com/content/bdb/paths/generate-tds-document.us.561144.pdf.
CD45RA (V450, Clone HI100, Cat. No. 560362, BD Biosciences) - antibody internally validated by gating on T cells from normal donor PBMCs as a positive control and fluorescence minus one negative controls; Vendor validation and technical information can be found at https://www.bdbiosciences.com/content/bdb/paths/generate-tds-document.us.560362.pdf.
CD45RO (APC-H7, Clone UCHL1, Cat. No. 561137, BD Biosciences) - antibody internally validated by gating on T cells from normal donor PBMCs as a positive control and fluorescence minus one negative controls; Vendor validation and technical information can be found at https://www.bdbiosciences.com/content/bdb/paths/generate-tds-document.us.561137.pdf.
BTLA (PE, Clone J168-540, Cat. No. 558485, BD Biosciences) - antibody internally validated by gating on T cells and B cells from normal donor PBMCs as well as expanded TIL as a positive control and fluorescence minus one negative controls; Vendor validation and technical information can be found at https://www.bdbiosciences.com/content/bdb/paths/generate-tds-document.us.558485.pdf.
CD28 (PE-Cy7, Clone CD28.2, Cat. No. 560684, BD Biosciences) - antibody internally validated by gating on T cells from normal donor PBMCs as a positive control and monocytes as a negative control; Vendor validation and technical information can be found at https://www.bdbiosciences.com/content/bdb/paths/generate-tds-document.us.560684.pdf.
Perforin (FITC, Clone DG9, Cat. No. 11-9994-42, eBiosciences) - antibody internally validated by gating on expanded TIL and T cells and NK cells from normal donor PBMCs as a positive control and B cells as a negative control; Vendor validation and technical information can be found at https://www.thermofisher.com/antibody/product/Perforin-Antibody-clone-dG9-delta-G9-Monoclonal/11-9994-42.
Granzyme B (V450, Clone GB11, Cat. No. 561151, BD Biosciences) - antibody internally validated by gating on expanded TIL and T cells and NK cells from normal donor PBMCs as a positive control and monocytes as a negative control; Vendor validation and technical information can be found at https://www.bdbiosciences.com/content/bdb/paths/generate-tds-document.us.561151.pdf.
IFNg (PE, Clone B27, Cat. No. 559327, BD Biosciences) - antibody internally validated by gating on stimulated, expanded TIL and T cells from normal donor PBMCs as a positive control and unstimulated T cells from normal donor PBMCs as a negative control. Vendor validation and technical information can be found at https://www.bdbiosciences.com/content/bdb/paths/generate-tds-document.us.559327.pdf.

Multiplex immunofluorescence antibodies were previously validated as reported in https://pubmed.ncbi.nlm.nih.gov/29042640.

Human research participants

Policy information about studies involving human research participants

Population characteristics	Male and female patients who met inclusion criteria for the study were 18 years of age and older and had stage IB (equal to or greater than 4 cm) to IIIA NSCLC according to American Joint Commission on Cancer (AJCC) 7th edition staging system. Only single mediastinal ipsilateral N2 station was allowed for the enrollment. All patients had to have surgically resectable disease and Eastern Cooperative Group performance status 0-1, adequate organ function, and cardiopulmonary status. Patients were excluded from the study if they had autoimmune disease, immunodeficiency, or previously received immunotherapy for other disease, if they had active infectious disease requiring ongoing treatment or cancer within the last two years. A complete list of inclusion and exclusion criteria is included in the Methods of the manuscript. Patient characteristics, including self-reported sex, are reported in Table 1. Sex and/or gender was not considered in the trial design. Twelve females and ten males were recruited in the Nivo+CT arm; seven females and fifteen males were recruited in the Ipi +Nivo+CT arm. Ten and thirteen patients were less than 65 years of age in the Nivo+CT and in the Ipi+Nivo+CT arms, respectively; twelve and nine patients were more than 65 years of age in the Nivo+CT and in the Ipi+Nivo+CT arms, respectively. The participants were not compensated for their participation on the studies.
Recruitment	Patient enrollment of the nivolumab plus chemotherapy arm started on December 14, 2018 and ended on July 22, 2019 and followed by nivolumab plus ipilimumab plus chemotherapy arm which started on December 30, 2019 and ended on December 1, 2020. Patients were screened, enrolled and treated in the Departments of Thoracic/Head and Neck Medical Oncology and Thoracic Surgery at the University of Texas MD Anderson Cancer Center. The Thoracic Medical Oncology and Thoracic Surgery Clinics at MD Anderson Cancer Center implemented the same screening protocols to identify and offer enrollment to patients with medically operable/technically resectable NSCLC. Participants were informed about the clinical trial by the treating physicians and surgeons and the clinical trial coordination team before signing the consent.<br><br>Potential biases applicable to the study were the relatively subjective operability/resectability of the disease prior to enrollment and the self-selection bias, which could derive from patient health literacy about the study (clinicaltrial.gov). The impact of both biases on the results of our study were minimized by presenting and discussing eligible patients at multidisciplinary tumor board conference before enrollment and by the objective evaluation of all study endpoints.
Ethics oversight	Written informed consent was provided by all study participants or their legal representatives. The study was approved by the University of Texas MD Anderson Cancer Center's Institutional Review Board.

Note that full information on the approval of the study protocol must also be provided in the manuscript.

Clinical data

Policy information about clinical studies

All manuscripts should comply with the ICMJE guidelines for publication of clinical research and a completed CONSORT checklist must be included with all submissions.

Clinical trial registration	NCT03158129
Study protocol	Information regarding the study protocol can be found in the Methods of the manuscript and at https://clinicaltrials.gov/ct2/show/ NCT03158129.
Data collection	Patient enrollment of the nivolumab plus chemotherapy arm started on December 14, 2018 and ended on July 22, 2019 and followed by nivolumab plus chemotherapy with ipilimumab arm which started on December 30, 2019 and ended on December 1, 2020. Data were collected from commencement of the clinical study until reported data cut-off date (July 18, 2022) in the department of Thoracic/Head and Neck Medical Oncology and Thoracic Surgery at the University of Texas MD Anderson Cancer Center. Electronic clinical report forms were collected through the Data Management Initiative (DMI) project at the University of Texas MD Anderson Cancer Center and Microsoft Excel (v. 2016) Spreadsheets.
Outcomes	The primary endpoint of the trial was major pathologic response (MPR), defined as less than or equal to 10% viable tumor cells in the original resected tumor bed following neoadjuvant therapy on trial and assessed by the pathologists involved in the study as detailed in the methods of the manuscript. Select secondary endpoints included treatment toxicity, perioperative morbidity and mortality, quantification of CD8+ TILs in resected tissues, ORR, pCR, completeness of surgical resection, time-to-events (including EFS and OS), correlation of blood, tissue and stool biomarkers with efficacy. Exploratory endpoints included tissue-, blood-, stool- and imaging-based biomarkers. All outcomes were assessed by the study investigators using the methods and criteria detailed in the manuscript, including RECIST criteria v. 1.1, National Cancer Institute Common Terminology Criteria for Adverse Events v. 4., time-to-events (event-free survival [EFS] defined as the time from treatment initiation to any progression of primary lung cancer precluding planned surgery, any progression or recurrence (as assessed by imaging and/or histopathologically) of primary lung cancer after surgery, any progression of primary lung cancer in patients without surgery, or death from all causes, or to the time of last imaging), and overall survival [OS], defined as the time from treatment initiation to the time of death from all causes or to the time of last follow up, obituaries were cross-referenced for any unreported patient deaths), tissue scRNA-seq, tumor NanoString, tissue flow cytometry, tumor PD-L1 IHC, tissue multiplex immunofluorescence staining and 16S gut microbiome, by the study investigators.

Flow Cytometry

Plots

Confirm that:

☒ The axis labels state the marker and fluorochrome used (e.g. CD4-FITC).

☒ The axis scales are clearly visible. Include numbers along axes only for bottom left plot of group (a 'group' is an analysis of identical markers).

☒ All plots are contour plots with outliers or pseudocolor plots.

☒ A numerical value for number of cells or percentage (with statistics) is provided.

Methodology

Sample preparation	Fresh tumor tissue was disaggregated using a medimachine and subsequent filtering to generate a single cell suspension for staining. PBMCs were thawed, washed and resuspended for staining. Surface staining was performed in FACS Wash Buffer (lX DPBS with 1% Bovine Serum Albumin) for 30 min on ice using fluorochrome-conjugated monoclonal antibodies from BD Biosciences, Biolegend, and eBioscience. Cells were fixed in 1% paraformaldehyde solution for 20min at room temperature following surface staining. For panels containing transcription factors, cells were fixed and permeabilized using the BD Transcription factor kit according to the manufacturer's instructions. A complete list of the antibodies, catalog numbers, company and clones used are available. Dead cells were stained using AQUA live/dead dye (lnvitrogen)and excluded from the analysis.
Instrument	BD Fortessa X20 or Canto II (BD Bioscience)
Software	BD FACSDiva software v8.0.1. was used for data acquisition. FlowJo v. 10.5.3 was used for all flow cytometry analysis
Cell population abundance	No cells were sorted in this study.
Gating strategy	Cells were initially gated using FSC-A v SSC-A followed by singlet gates using SSC-Av SSC-H. Single cells were then gated for exclusion of dead cells. A QC metric of 100 events was required in the immediate parental gate for any subgating.

☒ Tick this box to confirm that a figure exemplifying the gating strategy is provided in the Supplementary Information.
